# The Nociceptor Primary Cilium Contributes to Mechanical Nociceptive Threshold and Inflammatory and Neuropathic Pain

**DOI:** 10.1523/JNEUROSCI.1265-24.2024

**Published:** 2024-09-30

**Authors:** Lindsey A. Fitzsimons, Larissa Staurengo-Ferrari, Eugen V. Khomula, Oliver Bogen, Dionéia Araldi, Ivan J. M. Bonet, Paul G. Green, Ethan E. Jordan, Finn Sclafani, Connor E. Nowak, Julie K. Moulton, Geoffrey K. Ganter, Jon D. Levine, Kerry L. Tucker

**Affiliations:** ^1^Deparment of Biomedical Sciences, College of Osteopathic Medicine, University of New England, Biddeford, Maine 04005; ^2^Center for Excellence in the Neurosciences, University of New England, Biddeford, Maine 04005; ^3^Department of Oral and Maxillofacial Surgery, UCSF Pain and Addiction Research Center, University of California San Francisco, San Francisco 94115; ^4^Department of Preventative and Restorative Dental Sciences, University of California San Francisco, San Francisco 94115; ^5^School of Biological Sciences, College of Arts and Sciences, University of New England, Biddeford, Maine 04005; ^6^Department of Medicine, Division of Neuroscience, University of California San Francisco, San Francisco 94115

**Keywords:** chemotherapy-induced neuropathy; Hedgehog, hyperalgesia, inflammation, nociceptor, primary cilium

## Abstract

The primary cilium, a single microtubule-based organelle protruding from the cell surface and critical for neural development, also functions in adult neurons. While some dorsal root ganglion neurons elaborate a primary cilium, whether it is expressed by and functional in nociceptors is unknown. Recent studies have shown the role of Hedgehog, whose canonical signaling is primary cilium dependent, in nociceptor sensitization. We establish the presence of primary cilia in soma of rat nociceptors, where they contribute to mechanical threshold, prostaglandin E_2_ (PGE_2_)-induced hyperalgesia, and chemotherapy-induced neuropathic pain (CIPN). Intrathecal administration of siRNA targeting *Ift88*, a primary cilium-specific intraflagellar transport (IFT) protein required for ciliary integrity, resulted in attenuation of *Ift88* mRNA and nociceptor primary cilia. Attenuation of primary cilia was associated with an increase in mechanical nociceptive threshold in vivo and decrease in nociceptor excitability in vitro, abrogation of hyperalgesia, and nociceptor sensitization induced by both a prototypical pronociceptive inflammatory mediator PGE_2_ and paclitaxel CIPN, in a sex-specific fashion. siRNA targeting *Ift52*, another IFT protein, and knockdown of NompB, the *Drosophila Ift88* ortholog, also abrogated CIPN and reduced baseline mechanosensitivity, respectively, providing independent confirmation for primary cilia control of nociceptor function. Hedgehog-induced hyperalgesia is attenuated by *Ift88* siRNA, supporting the role for primary cilia in Hedgehog-induced hyperalgesia. Attenuation of CIPN by cyclopamine (intradermal and intraganglion), which inhibits Hedgehog signaling, supports the role of Hedgehog in CIPN. Our findings support the role of the nociceptor primary cilium in control of mechanical nociceptive threshold and inflammatory and neuropathic pain, the latter Hedgehog-dependent.

## Significance Statement

Many neurons have a tiny antenna-like structure, the primary cilium, protruding from their cell body, which processes information about the extracellular environment as well as regulates intracellular signaling. We report experiments aimed at understanding the role of the primary cilium in pain sensory neurons (nociceptors), including in the setting of inflammatory and neuropathic pain. We establish that nociceptors bear a primary cilium and show that this organelle regulates detection of noxious stimuli and contributes to nociceptor sensitization, using both the rat and the fruit fly as model organisms to manipulate primary cilia in pain states. We also identify primary cilium dependence of the contribution of Hedgehog to pain states.

## Introduction

The primary cilium, a single plasma membrane protrusion present on many cell types, contains a microtubule-based axoneme extending from a centriole-based basal body, situated in the apical portion of the cytoplasm (Goetz and Anderson, 2010; Hoyer-Fender, 2013). Diverse clinical syndromes, “ciliopathies,” occur in patients with gene defects for proteins that localize to the cilium and its basal body (Badano et al., 2006; Reiter and Leroux, 2017). While changes in sensory neurons have been reported in patients with Bardet–Biedl ciliopathy (Tan et al., 2007), distinct pain syndromes that enhance or attenuate pain have not been described in patients with ciliopathies.

During embryonic development, shortly after elaborating axons, dorsal root ganglion (DRG) neurons display a primary cilium (Yusifov et al., 2021). While they are likely present in some DRG neurons in postnatal stages (Tan et al., 2007; Yusifov et al., 2021), it is not known if primary cilia are expressed by and functional in nociceptors. Given that the primary cilium modulates electrophysiological properties and gene transcription in hippocampal pyramidal neurons (Berbari et al., 2014), the following hypothesis can be formulated: primary cilia are expressed by adult nociceptors, in which they modulate activation threshold and contribute to inflammatory and neuropathic pain.

Binding of Hedgehog (Hh) ligands to the Patch1 receptor allows for translocation of the G-protein-coupled receptor Smoothened (Smo) into the primary cilium, where it activates Hh signaling (Corbit et al., 2005; Rohatgi et al., 2007). Importantly, primary cilia are essential for signal transduction of the canonical Hh pathway (Ho and Stearns, 2021), which has been implicated in nociceptor sensitization (Babcock et al., 2011; Liu et al., 2018a, b; L. Han et al., 2020; Okuda et al., 2022; Zheng et al., 2023). Recent studies have reported the role for Hh signaling in the transduction and processing of nociceptive signals in both *Drosophila* (Babcock et al., 2011; Follansbee et al., 2017; Gjelsvik et al., 2018; Brann et al., 2019; McParland et al., 2021) and rodents (Babcock et al., 2011; Liu et al., 2018a, b; L. Han et al., 2020; Moreau and Boucher, 2020; Yang et al., 2020), morphine-induced tolerance and hyperalgesia (Babcock et al., 2011; Liu et al., 2018b), bone (Liu et al., 2018a) and pancreatic (L. Han et al., 2020) cancer pain, and chronic post-thoracotomy pain (Yang et al., 2020; reviewed in Zheng et al., 2023). However, whether Hh signaling in nociceptors is primary cilium dependent remains to be established. In the present experiments in mammals and insects, we explored the role and evolutionary conservation of the primary cilium and cilium-dependent Hh signaling in baseline nociceptor function (i.e., setting mechanical nociceptive threshold) and inflammatory and neuropathic pain (i.e., modulating nociceptor sensitization).

## Materials and Methods

### Animals

#### Rat

All experiments using rats were performed on 300–400 g male or 240–320 g female Sprague Dawley rats (Charles River Laboratories) that were housed three per cage under a 12 h light/dark cycle in a temperature- and humidity-controlled room at the University of California, San Francisco animal care facility. Food and water were available *ad libitum* in home cages. Protocols for rat experiments were approved by the University of California, San Francisco, Institutional Animal Care and Use Committee (IACUC), and adhered to the National Institutes of Health (NIH) Guidelines for the care and use of laboratory animals.

#### Drosophila

Flies were obtained from the Bloomington *Drosophila* Stock Center (BDSC) and maintained on sucrose-cornmeal-yeast medium at 25°C, between 45 and 60% humidity with 12 h light/dark cycle, and analyzed within 4 h of arbitrary dawn.

### Immunohistofluorescence analysis of primary cilia in lumbar DRGs

Rats were anesthetized with 5% isoflurane followed by perfusion through the left ventricle with 100 ml of cold phosphate-buffered saline (PBS) containing 10 U heparin/ml PBS, followed by 300 ml of methanol-buffered 10% formalin (Thermo Fisher Scientific). L4 and L5 DRGs were dissected out, bilaterally, and transferred into PBS containing 30% sucrose and 0.02% NaN_3_. DRGs were mounted in red tissue freezing medium (Electron Microscopy Sciences) and 15 μm sections cut with a cryostat (Leica CM1900). Sections were then mounted on glass slides and used for immunohistofluorescence analyses.

Presence and length of primary cilia on DRG neurons were investigated using antibody staining of histological sections of adult rat DRGs and acutely dissociated cultures prepared from fresh surgically isolated rat DRGs. Slides containing either DRG sections or coverslips with DRG neuronal cultures were rehydrated and washed three times with 1× PBS, followed by a 30 min incubation in blocking buffer (1× PBS, 0.3% Triton X-100, 5% normal goat serum). Blocking buffer was then aspirated, and primary antibodies, in antibody incubation buffer (1× PBS, 0.3% Triton X-100, 1% bovine serum albumin), were applied and allowed to incubate in a humid, dark chamber overnight at 4°C. Slides were then allowed to equilibrate to room temperature, and primary antibody solution aspirated. Slides were then washed three times with 1× PBS and secondary antibodies in antibody incubation buffer applied and allowed to incubate at room temperature in a light excluding humid chamber for 45 min. Slides were then washed five times with 1× PBS, followed by one rinse in a Coplin jar containing DI H_2_0 and finally coverslipped with DAPI mounting medium (Abcam) before imaging with a confocal microscope. The following primary antibodies were employed: rabbit anti-ARL13B (ADP-ribosylation factor-like 13B; Proteintech) 1:1,000, mouse anti-ɣ-tubulin (clone GTU-88, Sigma-Aldrich) 1:1,000, goat anti-TrkA (tropomyosin receptor kinase A; R&D Systems) 1:300, rabbit anti-ACIII (adenylyl cyclase III; EnCor) 1:4,000, mouse NeuN (RNA binding fox-1 homolog 3; Proteintech) 1:1,000, goat anti-rat calcitonin gene-related peptide (CGRP; Bio-Rad) 1:1,000, and Alexa 647-conjugated IB4 (isolectin GS-IB_4_; Invitrogen Life Technologies) 1:500. The following secondary antibodies were employed: goat anti-rabbit IgG (H + L; Alexa 488-conjugated, Invitrogen) 1:1,000, goat anti-mouse IgG (H + L; Alexa 555-conjugated, Invitrogen) 1:1,000, and rabbit anti-goat IgG (H + L; Alexa 647-conjugated, Invitrogen) 1:1,000.

### Imaging and data processing

Microscopy was conducted with a Leica Stellaris laser scanning confocal microscope utilizing Leica LAS X software, version 4.6, and Plan Apo 40×/1.3 NA and 63×/1.4 NA objectives. Lasers that are 405, 488, 561, and 638 nm with appropriate beam splitters (substrate or TD 488/561/633) were activated and emission ranges selected for each fluorophore. Sequential scanning between frames was then activated at 1,024 × 1,024 resolution using frame average = 2. The channel containing the ciliary marker was then used to establish the appropriate *z*-volume. Optimal step size was established using the Nyquist Sampling Theorem calculated by LAS X. A *z*-stack of interest was then collected, titled, and saved for processing. Data was transferred as .lif files from the confocal software to FIJI. Channels were split individually and stacks projected to maximum intensity. The lookup table (LUT) was set to grayscale for each of the channels to adjust brightness and contrast. LUTs were then set for cell nuclei in blue, cilia in green, basal body in red, and grayscale or yellow for neuronal markers.

### Culturing rat DRG neurons for immunohistofluorescence analysis and electrophysiology

Primary cultures of DRGs were made from 220–235 g adult male Sprague Dawley rats (Charles River Laboratories), as described previously (Araldi et al., 2018; Khomula et al., 2019; Bonet et al., 2021; Khomula et al., 2021). Under isoflurane anesthesia, rats were decapitated, and the dorsum of their vertebral column surgically removed. L4 and L5 DRGs were then rapidly extirpated, bilaterally, chilled, and desheathed in Hanks balanced salt solution (HBSS), on ice. DRGs were treated with 0.25% collagenase Type 4 (Worthington Biochemical) in HBSS for 18 min at 37°C, and then with 0.25% trypsin (Worthington) in calcium- and magnesium-free PBS (Invitrogen) for 6 min, followed by three washes and trituration in Neurobasal-A medium (Invitrogen) to produce a single-cell suspension. This suspension was centrifuged at 1,000 rpm for 3 min followed by resuspension in Neurobasal-A medium supplemented with 50 ng/ml nerve growth factor, 100 U/ml penicillin/streptomycin, B-27, GlutaMAX, and 10% FBS (Invitrogen). Cells were then plated on coverslips and incubated at 37°C in 3.5% CO_2_ for at least 24 h before they were used in patch-clamp electrophysiology experiments. One hour after plating, 1 ml of culture media was gently added. The next day another 1 ml of culture media was added. Cultured neurons were used in electrophysiology experiments within 24–72 h after preparation.

Each of three rats in experimental (*Ift88* siRNA-treated) group received three intrathecal injections of 10 μg *Ift88* siRNA on 3 consecutive days. Cultures of DRG neurons from these rats were prepared on Day 4 after the last injection. At UCSF, eight coverslips were prepared from each rat: six coverslips of 12 mm diameter for confocal microscopy (90 µl of cell suspension per coverslip) and two coverslips of 15 mm diameter for in vitro electrophysiology (120 µl of cell suspension per coverslip).

Coverslips designated for confocal microscopy were fixed by adding 2 ml of methanol-buffered 10% formalin (Thermo Fisher Scientific) to Petri dishes for 5 min, 72 h after preparation, then washed twice with PBS, and placed into individual wells of four-well plates. Each well was filled with PBS to the top, covered with a clean coverslip, gently removing bubbles, and tightly sealed with parafilm. In this state, they were stored in the fridge at 4°C and then, after all groups were prepared, the whole set was shipped to the University of New England for analysis.

Coverslips designated for in vitro electrophysiology were fractured to obtain 6–8 individual pieces on the day after culture preparation. (Each piece was used to perform an experiment on one cell and then discarded.) Cultured neurons were used in electrophysiology experiments within 24–72 h after preparation. (Thus, patch-clamp electrophysiology in *Ift88* siRNA-treated DRG neurons were performed on Days 5–7 after the last injection, when near maximal effect of siRNA was expected.)

### Whole-cell patch-clamp electrophysiology

Following placement of individual coverslip fragments plated with cells from dissociated rat DRGs in the recording chamber, culture medium was replaced by the solution used to perform electrophysiology: Tyrode's solution containing (in mM) 140 NaCl, 4 KCl, 2 MgCl_2_, 2 CaCl_2_, 10 glucose, and 10 HEPES, adjusted to pH 7.4 with NaOH, with an osmolarity of 310 mOsm/kg. The recording chamber had a volume of 150 µl and its perfusion system a flow rate of 0.5–1 ml/min. Electrophysiology experiments were conducted at room temperature (20–23°C; Khomula et al., 2021).

Cells on the coverslips were identified as neurons by their double birefringent plasma membranes (Cohen et al., 1968; Landowne, 1993). Whole-cell patch-clamp recordings, performed in current clamp mode, were used to evaluate baseline threshold and for changes in excitability of cultured rat DRG neurons (diameter <35 μm). Holding current was adjusted to maintain membrane potential at −70 mV. Rheobase, defined as the minimum magnitude of a current step needed to elicit an action potential (AP), was used to measure neuronal excitability threshold (Khomula et al., 2019, 2021; Vasylyev et al., 2023). Rheobase was determined through a testing protocol utilizing sequential square wave pulses with current magnitude increasing by a constant step, until an AP was elicited. An initial estimate of rheobase was made with incremental steps of 500 pA (0.5–4 nA). The increments were then adjusted to achieve 5–10% precision of the rheobase estimate (Khomula et al., 2021).

Whole-cell patch-clamp recordings, performed in voltage-clamp mode, were used to evaluate magnitude of inward and outward current, at baseline, in the cultured rat small-diameter DRG neurons (diameter <35 µm). Magnitude of current was measured as current density defined as current normalized to membrane capacity. Inward current (fast and transient) was characterized by peak current density (current density at the negative peak of inward transient current). Outward current was characterized by average current density of “plateau” at the end of a 20 ms stimulus. Both inward and outward current were measured in the same experiment at different time points of the same biphasic current elicited by step depolarization from a holding potential of −70 to 0 mV.

The current–voltage dependence of the fast inward current was obtained as dependence of peak current density on the potential of the depolarizing step (from a holding of −70 mV) in a series of stimulations with the potential of depolarizing step increasing from −50 to +20 mV with 5 mV increments. In this experiment the following parameters were measured: potential of depolarizing step at which peak current density was maximal, half-activation potential (potential at which peak current density was 50% of its maximal value), and the degree of activation (% of maximum) at −40 mV.

Recording electrodes were fashioned from borosilicate glass capillaries (0.84/1.5 mm inside diameter/outside diameter, Warner Instruments) using a Flaming/Brown P-87 microelectrode puller (Sutter Instrument). After being filled with a solution containing (in mM) 130 KCl, 10 HEPES, 10 EGTA, 1 CaCl_2_, 5 MgATP, and 1 Na-GTP, pH 7.2 (adjusted with Tris-base), resulting in 300 mOsmol/kg osmolarity, recording electrode resistance was ∼2 MΩ. Junction potential was not adjusted. Series resistance was below 10 MΩ, measured at the end of the recording, without compensation. Recordings were conducted using an Axon MultiClamp 700B amplifier, filtered at 20 kHz, and sampled at 50 kHz through an Axon Digidata 1550B controlled by pCLAMP 11 software (all from Molecular Devices). Drugs were applied at least 5 min after the establishment of whole-cell configuration to ensure the stability of a cell's baseline current.

### RNA extraction and reverse transcription quantitative real-time polymerase chain reaction

Total RNA from rat L4 and L5 DRG was extracted using Trizol (Invitrogen) and the PureLink RNA Mini Kit (Invitrogen) according to the manufacturer's instruction. The RNA concentration in each sample was determined with a spectrophotometer (Shimadzu). RNA was transcribed into cDNA using the iScript Advanced cDNA Synthesis Kit for RT-qPCR (Bio-Rad). Real-time PCR (polymerase chain reaction) was performed with the SsoAdvanced Universal SYBR Green Supermix (Bio-Rad) and specific primers for *Ift88* (intraflagellar transport protein 88; Bio-Rad, assay ID: qRnoCID0004310) and *Ift52* (Bio-Rad assay ID: qRnoCID0002245) on the CFX 96 real-time PCR detection system (Bio-Rad). The PCR program was run with the following conditions: 2 min at 95°C, followed by 40 cycles of 15 s at 95°C, and 30 s at 60°C. Each sample was run in triplicate. Change in gene expression was determined by the 2^−ΔΔCT^ method and expressed as relative change with respect to control level. Glycerinaldehyde-3-phosphate dehydrogenase (GAPDH) was used as a reference gene (Bio-Rad, assay ID: qRnoCID0057018).

### Behavioral pharmacology and patch-clamp electrophysiology reagents

#### Behavioral pharmacology

Prostaglandin E_2_ (PGE_2_), cyclopamine hydrate, and paclitaxel (purified from *Taxus yunnanensis*) were purchased from Sigma-Aldrich. Recombinant rat Sonic hedgehog (Shh) was purchased from Abcam. Stock solutions of PGE_2_ (1 μg/μl) in absolute ethanol was diluted to 20 ng/μl with 0.9% NaCl immediately before injection. The ethanol concentration of the final PGE_2_ solution was ∼2% and the injection volume 5 μl. Paclitaxel was prepared in absolute ethanol and polyethoxylated castor oil (Cremophor EL; 1:1; Sigma-Aldrich) and further diluted in saline, to a final concentration of 1 mg/ml. Cyclopamine hydrate was diluted in 0.9% NaCl containing 2% DMSO and was administered by intradermal (i.d.) or intraganglion (i.gl.) routes of administration, both 10 μg. Recombinant Shh was reconstituted in 0.9% NaCl at 0.1 mg/ml and further diluted in 0.9% NaCl to be administered in intradermal or intraganglion injection at 200 ng/animal. All doses were selected based on previous studies that established effectiveness at their targets (Ferrari et al., 2010; Liu et al., 2018a; Staurengo-Ferrari et al., 2022, 2023).

#### Patch-clamp electrophysiology (drugs and media)

The following reagents were used in the electrophysiology experiments: prostaglandin E_2_ (PGE_2_), NaCl, KCl, MgCl_2_, CaCl_2_, NaOH, MgATP, Na-GTP, D-Glucose, 4-(2-hydroxyethyl)piperazine-1-ethanesulfonic acid (HEPES) and ethylene glycol-bis(2-aminoethylether)-*N*,*N*,*N′*,*N′*-tetra-acetic acid (EGTA; Sigma-Aldrich), collagenase type 4, trypsin (Worthington), calcium- and magnesium-free HBSS, calcium- and magnesium-free PBS, Neurobasal-A medium, B-27 supplement, GlutaMAX, fetal bovine serum (FBS) and rat (recombinant) nerve growth factor (NGF)-beta (Invitrogen); IFT88 siRNA (Thermo Fisher Scientific, siRNA ID number S157132), mIFT52 siRNA (Thermo Fisher Scientific, siRNA ID number S187205), and normal control (nc) siRNA (Thermo Fisher Scientific).

Stock solution of PGE_2_ was prepared in absolute ethanol (1 mg/ml, 2.83 mM) and stored as tightly sealed 10 µl aliquots at −80°C. For in vitro electrophysiology, a secondary stock solution of PGE_2_ was prepared in purified water (25 µM, 1:112 dilution) on the day of the experiment and stored at 4°C before. The final concentration of PGE_2_ in the perfusion solution was selected to be 100 nM, which produces moderate sensitization in vitro (Khomula et al., 2021), and was achieved by a 1:249 dilution of the secondary stock solution, in Tyrode's solution, performed just before it was used in experiments.

### Intrathecal administration of siRNA

Intrathecal (i.t.) administration of small interfering (si) RNA was used to silence the mRNA expression of *Ift88* (mIFT88, Thermo Fisher Scientific, Ambion in vivo predesigned siRNA, siRNA ID number S157132) and *Ift52* (mIFT52, Thermo Fisher Scientific Ambion in vivo predesigned siRNA pn4404010, siRNA ID number S187205) in vivo. A scrambled sequence was used as the negative control (nc-siRNA, Thermo Fisher Scientific, Ambion in vivo predesigned negative control siRNA). siRNAs were mixed with PEI (in vivo-jetPEI; Polyplus-transfection) according to the manufacturer's instructions. The N:P ratio (number of nitrogen residues of in vivo-jetPEI per DNA phosphate) used was 8:1 (1 μg of siRNA was mixed with 0.16 μl of in vivo-jetPEI). siRNA targeting *Ift88*, *Ift52*, and nc-siRNA were administered intrathecally for 3 consecutive days. To administer siRNAs, rats were briefly anaesthetized with 2.5% isoflurane, and a 30-gauge hypodermic needle was inserted into the subarachnoid space, on the midline, between the L4 and L5 vertebrae. The intrathecal site of injection was confirmed by the elicitation of a tail flick, a reflex that is evoked by accessing the subarachnoid space and bolus intrathecal injection (Mestre et al., 1994). This procedure produces not only reversible inhibition of the expression of the relevant proteins in DRG but also modulation of nociceptive behavior (Staurengo-Ferrari et al., 2023).

### Intraganglion administration of pharmacological agents

Intraganglion injection of compounds targeting Hh signaling into the L5 DRG was performed with a gingival needle (30-gauge), prepared as previously described (Ferrari et al., 2007; Araldi et al., 2013), attached to a 50 μl Hamilton syringe by a short length of PE-10 polyethylene tubing; modification of the injecting needle decreases the risk of tissue damage when its tip penetrates the DRG. Rats were lightly anesthetized by inhalation of isoflurane, and the fur over the lower back was shaved. The site of skin penetration was 1.5 cm lateral to the vertebral column and ∼0.5 cm caudal from a virtual line passing between the rostral edges of the iliac crests. To facilitate the insertion of the injecting needle through the skin, an initial cutaneous puncture was made with a larger (16-gauge) hypodermic needle. In sequence, the injecting needle was inserted through the puncture wound in the skin and oriented toward the region of the intervertebral space between the fifth and sixth lumbar vertebrae, until it touched the lateral region of the vertebrae. To reach the space between the transverse processes of the fifth and sixth lumbar vertebrae, small incremental movements of the needle tip were performed, until the resistance provided by the bone was diminished. When the tip of the needle reached the correct position, it felt “locked in place” (through the intervertebral space) and a flinch of the ipsilateral hindpaw was observed (Araldi et al., 2013), indicating that it had penetrated the DRG of the fifth lumbar spinal nerve (Ferrari et al., 2007). The procedure, starting from the beginning of anesthetic inhalation, until withdrawal of the injection needle, takes ∼3 min. Animals regained consciousness ∼2 min after anesthesia was discontinued. Importantly, no change in the nociceptive mechanical paw withdrawal threshold of the ipsilateral hindpaw, after a single injection or daily repeated injections in the L5 DRG, was observed, and immunofluorescence analysis of the L5 DRG after injections shows no signs of cell damage (Araldi et al., 2013). Likewise, no significant change in the mechanical nociceptive threshold in the hindpaw, after intraganglion injection of vehicle (single or repeated) was observed, indicating a lack of injection-induced damage (Araldi et al., 2013). This intraganglion injection technique has been used successfully by us and others (Araldi et al., 2013; Souza et al., 2013; Ferrari et al., 2014, 2015; Lemes et al., 2018; Teixeira et al., 2019; Neves et al., 2020).

### Intradermal administration of membrane-impermeable pharmacological agents

For the intradermal injection of cyclopamine, an inhibitor of the Shh signaling pathway, hypotonic shock (2 μl of distilled water, separated in the syringe by an air bubble) preceded their injection, to facilitate entry of membrane-impermeable compounds into nerve terminals (Keeney and Linn, 1990; Lepers et al., 1990; Schulz, 1990) on the dorsum of the hindpaw. In these experiments, a total volume of 5 μl was injected intradermally on the dorsum of the rat's hindpaw.

### Measuring mechanical nociceptive threshold

Mechanical nociceptive threshold in rats was quantified using an Ugo Basile Analgesy-meter (Stoelting), to perform the Randall–Selitto paw withdrawal test on the rat's hindpaw (Randall and Selitto, 1957; Taiwo et al., 1989; Taiwo and Levine, 1989). This device uses a dome-shaped plunger to apply a mechanical force to the dorsum of the hindpaw, which increases linearly with time. Rats were placed into cylindrical acrylic restrainers with lateral ports that allow access to the hindpaw, as previously described (Araldi et al., 2019), to acclimatize them to the testing procedure. Mechanical nociceptive threshold is defined as the force in grams at which a rat withdraws its paw. Baseline threshold is defined as the mean of three readings taken before injection of test agents; each experiment was performed on different groups of rats. Data are presented as mechanical nociceptive threshold (in grams) and as percentage change from preintervention baseline (baseline value minus postintervention value, and this difference then divided by the baseline value, for each paw).

### Paclitaxel chemotherapy-induced peripheral neuropathy (CIPN)

Using an established rat model of taxane-induced CIPN, paclitaxel was injected intraperitoneally (1 mg/kg × 4) every other day for a total of four doses; control animals received the same volume of vehicle with proportional amounts of Cremophor EL and ethanol diluted in saline (Staurengo-Ferrari et al., 2022; Bonet et al., 2023). The presence of paclitaxel-induced painful peripheral neuropathy (CIPN) was confirmed by a decrease in mechanical nociceptive threshold in the Randall–Selitto paw withdrawal test (Dina et al., 2001; Alessandri-Haber et al., 2004; Ferrari et al., 2020; Staurengo-Ferrari et al., 2022).

### PGE_2_-induced mechanical hyperalgesia

The pronociceptive inflammatory mediator, PGE_2_ (100 ng, i.d.), was injected on the dorsum of the rat's hindpaw at the site of nociceptive threshold testing, in a volume of 5 µl, using a 30-gauge hypodermic needle attached to a microsyringe (Hamilton). Hyperalgesia was quantified as the percentage decrease in mechanical nociceptive threshold, induced by PGE_2_. The dosing protocol and timing of mechanical nociceptive threshold assessment were based on previous studies (Ferrari et al., 2010; Bogen et al., 2012).

### *Drosophila* experiments

#### *Drosophila* genetics

Experimental genotype was the progeny of *ppk1.9-GAL4* (Ainsley et al., 2003) and *UAS-NompB-IR* (BDSC_28665)*.* Larvae of experimental genotype were compared with two controls; a “no Gal4” control consisting of the progeny of the Gal4 driver's background (*w^1118^*; BDSC_3605) crossed to the UAS-NompB-IR line and a “no UAS” control consisting of the progeny of the *ppk1.9-Gal4* driver line crossed to *y^1^v^1^* (BDSC_36303), the background of the UAS-NompB-IR line.

#### *Drosophila* immunohistofluorescence analysis

Third instar *Drosophila melanogaster* larvae [ppk1.9-tdTomato/nompB^MI08675^ (BDSC_44994, (Nagarkar-Jaiswal et al., 2015)] were filleted, pinned flat, and promptly fixed by 60 min incubation at room temperature (RT) with ice-cold 4% paraformaldehyde in PBS. Fixation was followed by two 1 min washes, one 10 min wash, and one 1 h wash at RT in PBT (PBS with 0.3% Triton X-100). Washed fillets were then blocked using PBT-B (PBT with 1% bovine serum albumin) for at least 1 h at RT. Fillets were incubated overnight at 4°C using gentle rotation with chicken anti-GFP (Antibodies) 1:1,000 in PBT-B, followed by two 30 min washes in PBT-B with rotation and then blocked for 1 h using fresh PBT-B + 5% normal donkey serum (NDS) at RT. Fillets were incubated for 2 h at RT with a donkey anti-chicken secondary antibody (AlexaFluor-488 conjugated, Jackson ImmunoResearch Laboratories), 1:500 in PBT-B + 5% NDS. Fillets were then washed three times in PBT for 30 min, followed by two washes for 2 min with PBS. Fillets were mounted on slides using Vectashield Antifade Mounting Medium with DAPI (H-1200, Vector Laboratories) and stored in the dark at 4°C until imaging. Confocal imaging conditions were used as described above (see above, Imaging and data processing).

#### *Drosophila* mechanical nociception assays

Nociception was assayed in large foraging third instar larvae by von Frey stimulation at 2,346 kPA as previously described (Lopez-Bellido et al., 2019). The dorsal surface at the midline of abdominal segment A8 was stimulated by an operator blinded to genotype. Behavior was evaluated for 10 s for occurrence of larval nocifensive escape behavior, characterized as a 360° roll around the longitudinal body axis (Tracey et al., 2003). Response frequencies of groups of larvae were compared with each normal control by chi-square test.

### Experimental design and statistical analyses

#### Behavioral studies

Only one hindpaw per rat was used in behavioral experiments. Data are presented as percentage change from baseline mechanical nociceptive threshold. Repeated-measures one-way analysis of variance (ANOVA), followed by the Bonferroni’s post hoc multiple comparison test or Student's *t* test, was used for statistical analyses. Prism 8.0 (GraphPad Software) was used for the graphics and to perform statistical analyses; *p *< 0.05 was considered statistically significant. Data are presented as mean ± SEM.

#### Electrophysiology studies

Rheobase was measured before and 5 min after application of PGE_2_ to cultured nociceptors. Magnitude of the sensitizing effect of PGE_2_ was expressed as percentage reduction in rheobase [i.e., its value before PGE_2_ administration (baseline) was subtracted from its value after, then the difference was divided by pre-PGE_2_ baseline]. Two-sample unpaired two-tailed Student's *t* test was used to compare pre-PGE_2_ rheobase and PGE_2_-induced reduction in rheobase between control and *Ift88* siRNA-treated groups. Two-way ANOVA (factors were type of current and treatment) followed by Šídák's multiple-comparisons test was used to compare baseline (pre-PGE_2_) inward and outward currents between control and *Ift88* siRNA-treated groups.

Prism 10.2 (GraphPad Software) was used to generate graphics and perform statistical analyses; *p < 0.05* is considered statistically significant. Data are presented as mean ± SEM.

## Results

### Adult rat nociceptors elaborate primary cilia, in vivo and in vitro

**Figure 1.**
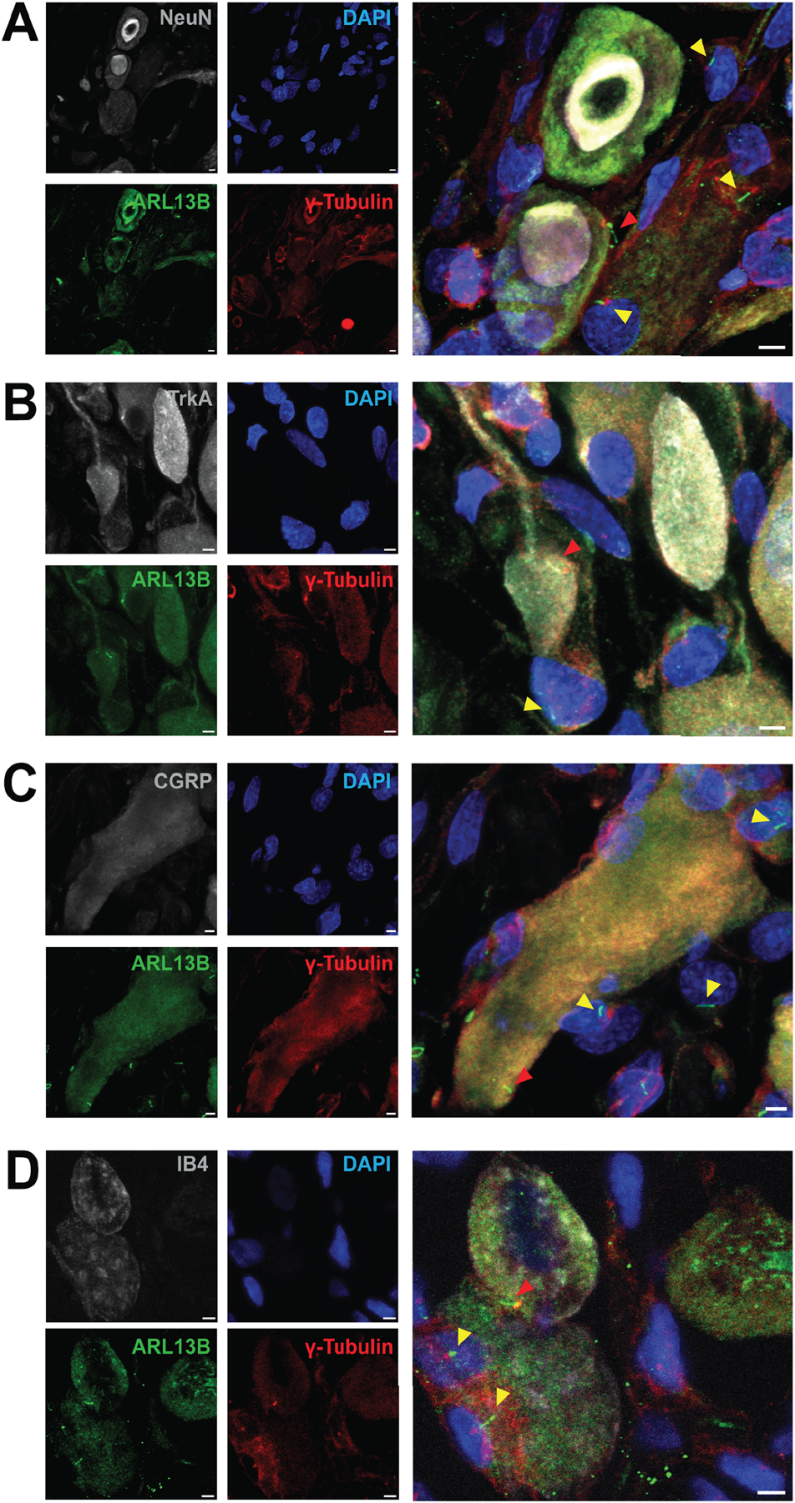
Rat DRG neurons elaborate primary cilia, in vivo. ***A–D***, Immunohistofluorescence analysis of adult rat DRGs in vivo, using confocal microscopy. Histological sections were labeled with antibodies recognizing ARL13B (***A–D***, green), ɣ-tubulin (***A–D***, red), and Fox-3 (***A***, NeuN, grayscale), TrkA (***B***, grayscale), or CGRP (***C***, grayscale). ***D***, IB4 staining is indicated in grayscale. ***A–D***, Cell nuclei are marked by DAPI (blue). Red and yellow arrowheads indicate neuronal and non-neuronal primary cilia, respectively. ***A–D***, Scale bar, 3 µm. See Extended Data Figure 1-1 for further cilium analysis.

**Figure 2.**
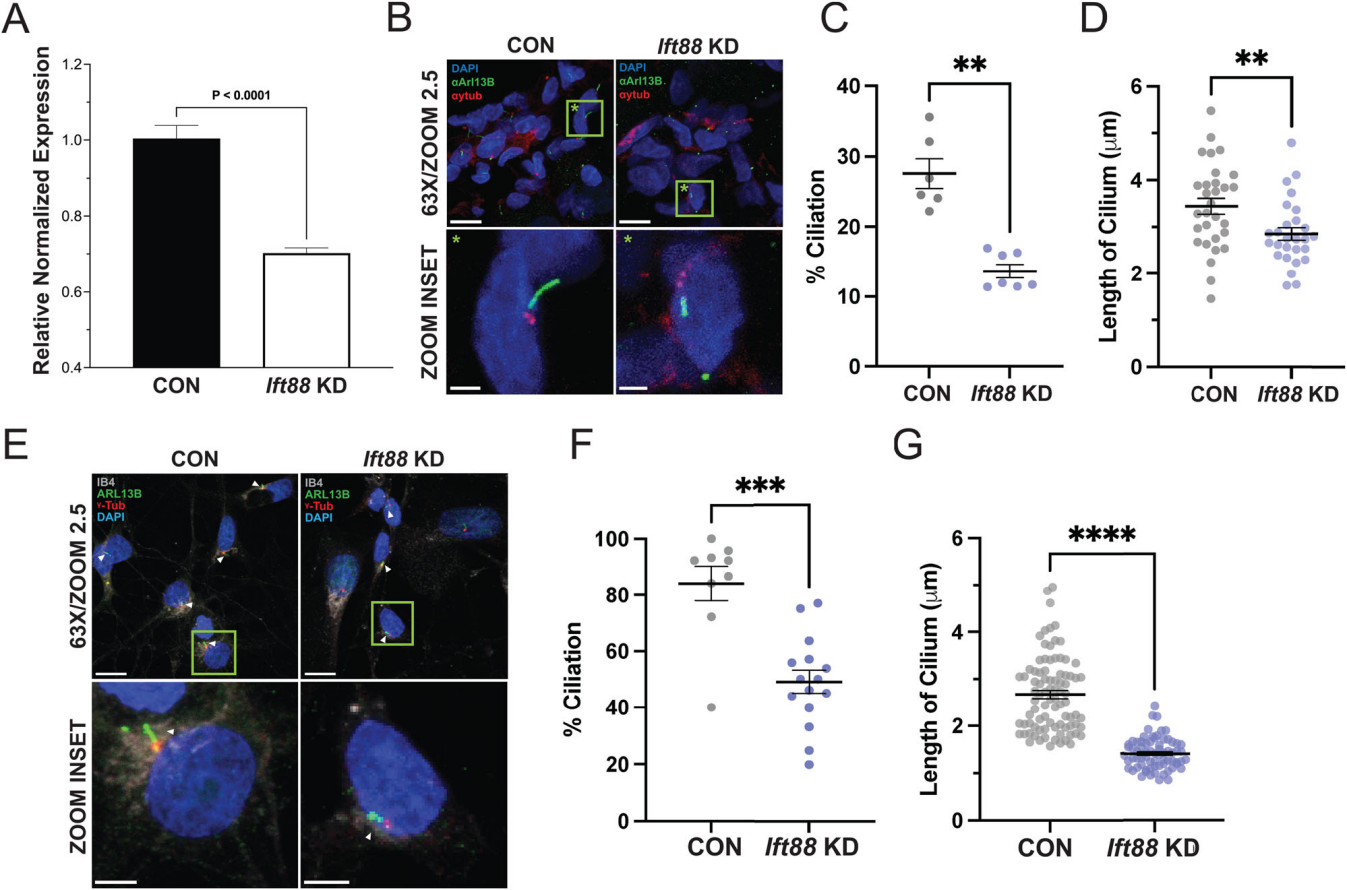
Targeted knockdown of *Ift88* in rat DRG leads to a loss of neuronal primary cilia and a decrease in the average length of retained cilia. ***A***, *Ift88* siRNA specifically reduces *Ift88* mRNA levels in the rat DRG. ***B–G***, Reduction in the fraction of ciliated cells and the length of primary cilia in DRG cells in vivo (***B–D***), and in ex vivo (***E–G***) acute cultures of DRG neurons exposed, in vivo, to siRNA targeting the rat *Ift88* gene, 7 d after final siRNA injection. ***B***, ***E***, Confocal immunofluorescence images of L4 rat DRGs stained for the ciliary axoneme (αArl13b, green), the basal body (αγtub, red), and the nucleus (DAPI, blue). Left panels, Control siRNA. Right panels, *Ift88*-targeted siRNA. Bottom panels, Magnifications of green boxes (green star) in the top panels. Magnification/zoom factor indicated. Scale bars: 10 µm, top panels; 2 µm, bottom panels. ***E***, IB4-stained cultured DRG neurons. Arrowheads indicate primary cilia. ***C***, ***D***, ***F***, ***G***, Quantification of primary cilium parameters. Percentage ciliation (***C***, ***F***) and cilium length (***D***, ***G***) in L4 rat DRGs injected with control versus *Ift88*-targeted (knockdown) siRNA, both (***C***, ***D***) in vivo and (***F***, ***G***) ex vivo neurons cultured from anti-*Ift88* siRNA-treated DRGs. **p < 0.01, ***p < 0.001, ****p < 0.00001.

Primary cilia were detected in adult rat DRG neurons using immunohistofluorescence staining with confocal microscopy. DRGs (Fig. 1*A–D*; Extended Data Fig. 1-1*B*) and cultures of neurons from acutely dissociated DRGs (Extended Data Fig. 1-1*A*) were labeled with an antibody recognizing the ciliary protein Arl13b (ADP-ribosylation factor-like 13B; Caspary et al., 2007), to identify the plasma membrane surrounding the central ciliary axoneme, and an antibody recognizing ɣ-tubulin to label the basal body from which the primary cilium grows (Schiebel, 2000; Ge et al., 2022). To identify the cells expressing these ciliary markers as neurons, we employed antibodies recognizing Fox-3/NeuN (Fig. 1*A*), TrkA (Fig. 1*B*), or calcitonin gene-related peptide (CGRP; Fig. 1*C*). We also identified neurons binding isolectin B4 (IB4), which labels nonpeptidergic nociceptors (Fig. 1*D*). In DRG cultures IB4 reactivity identified a population of nociceptors (Extended Data Fig. 1-1*A*, Fig. 2*E*). Primary cilia were found on many neurons in the rat DRG and elaborated by cultured rat DRG neurons. In DRGs, the large number of satellite cells surrounding neurons are also ciliated (Extended Data Fig. 1-1*B*), with cilia that appear to be oriented facing neuronal cell bodies.

10.1523/JNEUROSCI.1265-24.2024.f1-1 Figure 1-1 **Rat DRG neurons elaborate primary cilia *in vitro* and *in vivo.* (A, B)** Immunohistofluorescence analysis of acutely dissociated cultured adult rat DRG (**A**) and of adult rat DRGs *in vivo* **(B)**. Coverslips (**A**) and histological sections **(B)** were labeled with antibodies recognizing ARL13B (green), ɣ-tubulin (red), and Fox-3 (**B,** NeuN, greyscale). **(A)** IB4 staining is indicated in greyscale. **(A, B)** Cell nuclei marked by DAPI (blue). Red and yellow arrowheads indicate neuronal and non-neuronal primary cilia, respectively. Scale bar: 10  µm. Download Figure 1-1, TIF file.

### Intrathecal siRNA attenuates *Ift88* in rat DRG, associated with loss of primary cilia and decrease in the average length of retained cilia

Two classes of IFT protein complexes, B and A, are responsible for anterograde and retrograde trafficking of proteins into and out of the primary cilium, respectively (Prevo et al., 2017). ITF88 is an important component of the B complex (Rosenbaum and Witman, 2002). Primary cilia can be disrupted by the targeted inactivation of genes encoding proteins essential for maintenance of primary cilia structure, such as *Ift88*, an approach that we have previously employed (Willaredt et al., 2008; Willaredt et al., 2012; Gazea et al., 2016a, b). In the present experiments, male rats were treated with siRNA targeting *Ift88*, in a dose of 2, 5, or 10 μg/day, intrathecally (i.t.), for 3 consecutive days (Fig. 2*A–G*). Because rats treated with the highest dose of siRNA showed the strongest behavioral phenotype, L4 and L5 DRGs were harvested 7 d after the last injection of the 10 μg dose of *Ift*88 siRNA, from killed rats, to prepare mRNA, or rats were transcardially perfused with 4% paraformaldehyde to prepare tissue for immunohistofluorescence analysis. Total RNA, isolated from L4 and L5 lumbar DRGs, from rats treated with 10 μg siRNA or 10 μg of negative control (nc) siRNA, was used to generate cDNA through reverse transcription. Quantitative RT-PCR, performed on the resultant cDNA, revealed a significant decrease in *Ift88* in animals treated with the 10 μg dose of siRNA targeting *Ift88* (Fig. 2*A*). To visualize and quantify the primary cilium at the single-cell level, DRGs were harvested from paraformaldehyde-perfused rats, 7 d after the final injection of the 10 μg dose of siRNA targeting *Ift88*. Then 15-μm-thick sections of cryo-embedded L4 and L5 DRGs were labeled with antibodies recognizing Arl13b and ɣ-tubulin, followed by confocal microscopy (Fig. 2*B*). Counting of cells with and without cilia and length measurement of remaining cilia revealed a significant decrease in both the prevalence of cilia in DRG neurons (Fig. 2*C*) as well as the average length of remaining cilia (Fig. 2*D*), in animals treated with siRNA targeting *Ift88*, compared with rats treated with a negative control (nc) siRNA. In additional groups of siRNA and nc-siRNA-treated rats, L4 and L5 DRGs were harvested and cultured on coverslips for confocal microscopy. To specifically examine the ciliation status of putative nociceptive neurons, to capture the entirety of the neuronal soma and its cilium in the *z*-axis, we further prepared ex vivo cultures from rats treated for 3 d with anti-*Ift88* siRNAs, and 4 d later, DRGs were harvested. Neuronal cultures from acutely dissociated DRG were fixed after 3 d in culture and examined with the same immunohistofluorescence technique. Restriction of analysis to IB4-positive, small-diameter (soma diameter <35 µm) neurons (Fig. 2*E*) indicated a similar reduction in both ciliation (Fig. 2*F*) and cilium length (Fig. 2*G*).

### Effect of siRNA targeting *Ift88* on baseline mechanical nociceptive threshold, nociceptor excitability (rheobase), and hyperalgesia associated with paclitaxel CIPN

**Figure 3.**
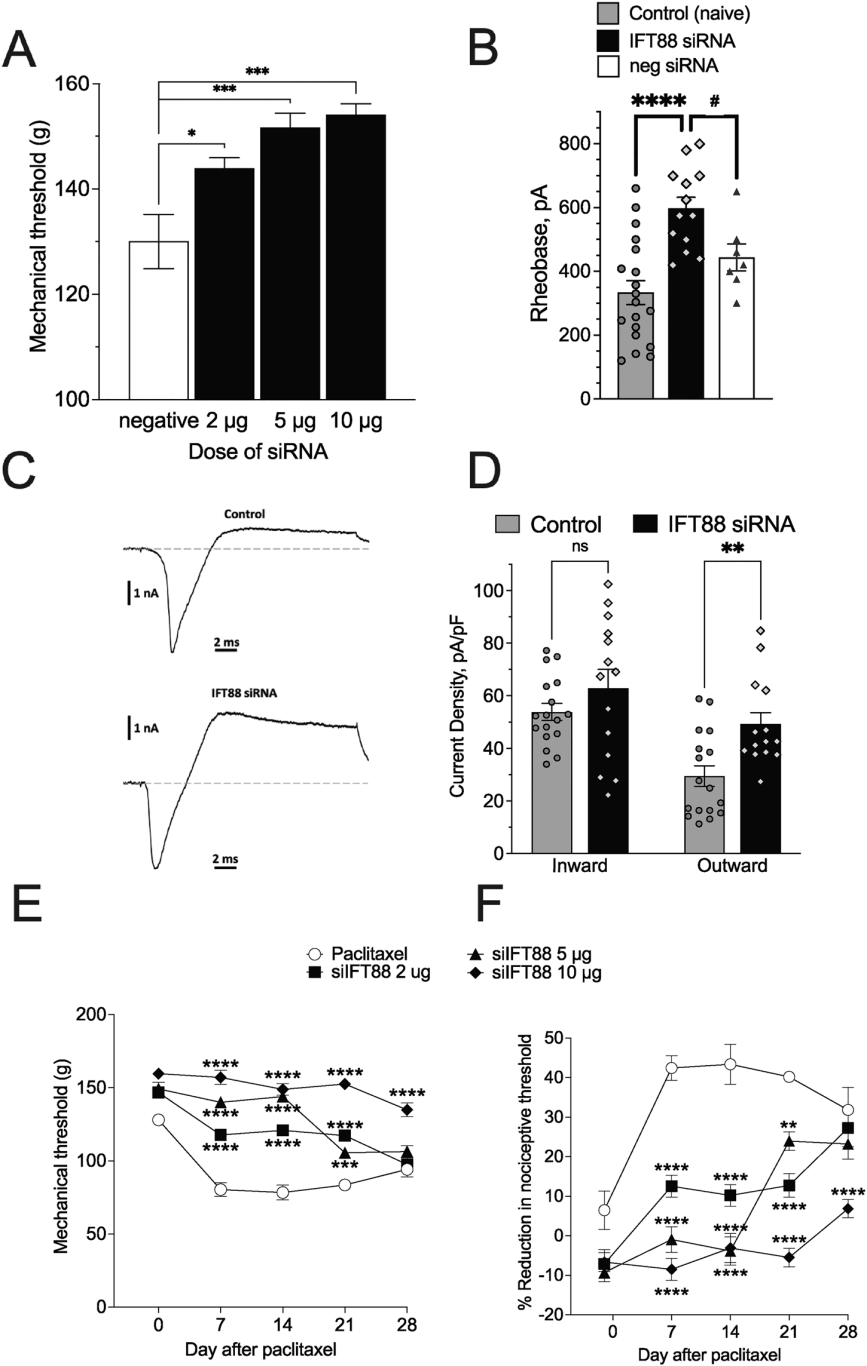
Effect of siRNA targeting *Ift88* on baseline mechanical nociceptive threshold, nociceptor rheobase, and paclitaxel CIPN. ***A*,** Male rats were treated intrathecally for 3 d with siRNA targeting *Ift88* or its negative control siRNA. Mechanical nociceptive threshold (presented in grams) was evaluated before and 72 h after the last siRNA treatment. At this time, nociceptive threshold was significantly increased by 2, 5, and 10 µg *Ift88* siRNA compared with negative control siRNA (*p *< 0.02, 0.0005, and 0.0001, respectively). ***B***, *Ift88*-targeting siRNA administered in vivo produced a significant elevation in rheobase in nociceptors cultured from *Ift88* siRNA-treated rats, compared with rheobase in nociceptors cultured from control and negative siRNA-treated rats (****p < 0.0001, *t*_(36) _= 5.1; ^#^*p *= 0.03, *t*_(36) _= 2.3, Šidák's multiple-comparisons test for ANOVA; effect of treatment is significant: *p *< 0.0001, *F*_(2,36) _= 12.0; *n *= 19 control, 13 *Ift88* siRNA-treated and 7 negative siRNA-treated groups). ***C***, Example traces of depolarization-induced whole-cell current in rat small-diameter (<35 µm) DRG neurons cultured from control (naive) and in vivo *Ift88* siRNA-treated rats. The stimulus current was elicited by step depolarization from a holding potential of −70 to 0 mV for 20 ms using voltage-clamp mode of the whole-cell patch-clamp configuration. The biphasic current with rapid-onset inward phase (driven by sodium voltage-gated ion channels) transitioning into a slower outward phase (driven by potassium voltage-gated ion channels). ***D***, Peak current density of the inward (magnitude; current is negative) and outward phases (positive, measured at the end of depolarization). While there was no significant change in inward current, the outward current was upregulated by 63% (*p = 0.011, *t*_(60) _= 2.9, Šidák's multiple-comparisons test for two-way ANOVA; effect of treatments is significant: *p *= 0.004, *F*_(1,60) _= 8.9). ***E***, ***F***, Alleviation of CIPN hyperalgesia by intrathecal administration of *Ift88* siRNA. Seventy-two hours after the last administration of anti-Ift88 siRNA (***A***), paclitaxel (1 mg/kg, i.p.) was administered (Days 0, 2, 4, and 6). Mechanical nociceptive threshold was evaluated before siRNA treatment was started (baseline) and again on Days 7, 14, 21, and 28 after the last dose. siRNA treatment significantly attenuated CIPN hyperalgesia (two-way repeated-measures ANOVA; ***E***, *p *< 0.0001, *F*_(9,80) _= 11.23; ***F***, *p *< 0.0001, *F*_(9,80) _= 11.37 followed by Bonferroni’s multiple-comparisons test: **p < 0.01; ***p < 0.005; ****p < 0.0001, *n* = 6 paws of each group).

Male rats were treated with siRNA targeting *Ift88* (2, 5 or 10 μg/day, i.t.) or negative control (nc) siRNA, for 3 consecutive days. Seventy-two hours after the last administration of siRNA, when mechanical nociceptive threshold was evaluated by the Randall–Selitto paw withdrawal test, threshold was significantly increased by all three doses of *Ift88* siRNA, when compared with rats receiving the same dose of the negative control (nc) siRNA (Fig. 3*A*).

To test the hypothesis that primary cilia regulate the biophysical properties of nociceptors underlying the elevated mechanical nociceptive threshold in *Ift88* siRNA-treated rats, we performed in vitro patch-clamp electrophysiology in DRG neurons cultured acutely from rats treated in vivo with *Ift88* siRNA administered in vivo on neuronal excitability. Rheobase, a well-defined electrophysiological property that reflects excitability threshold in DRG neurons (Khomula et al., 2019, 2021; Vasylyev et al., 2023), was selected as our measure of neuronal excitability. In putative C-type nociceptive DRG neurons (soma diameter <35 µm; Harper and Lawson, 1985; Gold et al., 1996; Petruska et al., 2002; Woolf and Ma, 2007), rheobase in the *Ift88* siRNA-treated nociceptors was significantly greater than from nociceptors in negative control siRNA-treated rats (by 79%; unpaired two-tailed Student's *t* test: ****p < 0.0001, *t*_(30) _= 4.9; Fig. 3*B*) or in naive rats (Fig. 3*B*), indicating an increase in action potential threshold. Input (membrane) resistance in control (100 ± 6 MOhm) and after *Ift88* knockdown (92 ± 7 MOhm) were not significantly different (unpaired two-tailed Student's *t* test: *p* = 0.43, *t*_(26) _= 0.80, *n* = 14 per group), thus unlikely contributing to the larger rheobase in the *Ift88* siRNA-treated group.

The increase in rheobase produced by treating rats with *Ift88* siRNA was associated with an increase in peak outward current, with no significant change in peak fast inward current (Fig. 3*C*,*D*; two-way ANOVA, effect of treatment: *F*_(1,58) _= 9.3, *p* = 0.004; Šídák's multiple-comparisons test: for outward current *t*_(1,58) _= 3.0, **p = 0.009; for inward current *t*_(1,58) _= 1.3, ns, *p* = 0.34). A further analysis of the current–voltage dependence of the fast inward current also did not reveal changes in half-activation potential, the potential at which peak current was maximal, and the degree of activation (% of maximal magnitude) at −40 mV (around AP threshold; data not shown). While we cannot absolutely exclude indirect effects mediated by primary cilia on non-neuronal cells in our DRG neuron cultures, we believe this to be highly unlikely since we used a low cell culture density, with cells separated by >100 µm, which would produce an estimated dilution of intercellular mediators of 4 orders of magnitude.

We next employed rats treated with paclitaxel, to evaluate the role of the primary cilium in a preclinical model of neuropathic pain. A group of male rats were treated with *Ift88* siRNA (2, 5, or 10 μg/day, i.t.) or a negative control siRNA for 3 consecutive days. Seventy-two hours after the last administration of siRNA, paclitaxel (1 mg/kg, i.p.) was administered (Days 0, 2, 4, and 6). Mechanical nociceptive threshold was evaluated before siRNA treatment was started (baseline), and again on Days 7, 14, 21, and 28 after the last dose of paclitaxel, with the highest dose of *Ift88* siRNA bringing animals essentially back to baseline values, as compared with rats receiving an intrathecal dose of the negative control siRNA (Fig. 3*E*,*F*).

### Effect of siRNA targeting *Ift52* on paclitaxel CIPN

**Figure 4.**
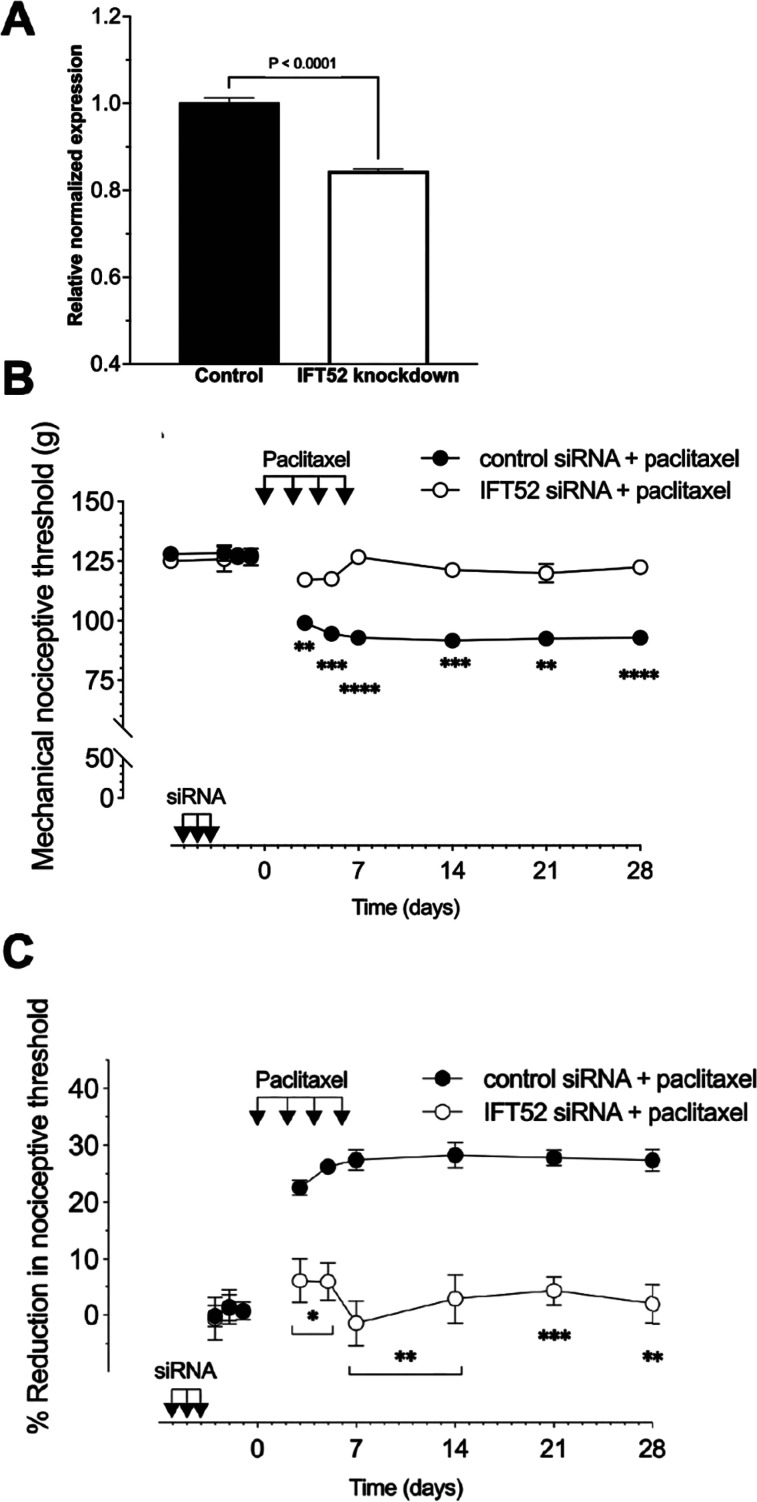
Effect of siRNA targeting *Ift52* on paclitaxel-induced mechanical hyperalgesia (CIPN). ***A***, siRNA-mediated knockdown of *Ift52* mRNA. The histogram depicts the level of *Ift52* mRNA in DRG from rats treated with either siRNA targeting *Ift52* or control siRNA. Gene expression was determined with SYBR green RT-PCR and values (mean ± 95% CI) represent expression relative to GAPDH (the housekeeping gene). Rats treated with siRNA targeting *Ift52* demonstrate a significant reduction in *Ift52* mRNA levels in their lumbar DRG compared with those treated with the control siRNA (unpaired two-tailed Student's *t* test; *p *< 0.0001; *t*_(10)_ = 11.21; *n* = 6). ***B***, ***C***, Male rats were treated with siRNA targeting *Ift52* (10 µg/day, i.t.) for 3 consecutive days. Seventy-two hours after the last siRNA injection, paclitaxel (1 mg/kg, i.p.) was administered, Days 0, 2, 4, and 6. Mechanical nociceptive threshold was evaluated before siRNA treatment was started (baseline), and again on Days −3, −2, −1, 3, 5, 7, 14, 21, and 28 d after paclitaxel injection. ***B***, Magnitude of hyperalgesia is expressed as absolute values of mechanical nociceptive threshold, in grams. *Ift52* siRNA attenuates paclitaxel induced CIPN. Data are expressed as mean ± SEM, *n *= 6 paws in each group. *F*_(9,90) _= 29.38, ****p < 0.0001, ***p < 0.0002, **p < 0.0028; when *Ift52* siRNA group was compared with control siRNA group; two-way repeated-measures ANOVA followed by Bonferroni’s multiple-comparisons test. ***C***, Hyperalgesia magnitude is expressed as percentage reduction from the baseline mechanical nociceptive thresholds. *Ift52* siRNA attenuates paclitaxel induced CIPN. Data are expressed as means ± SEM, *n *= 6 paws in each group. *F*_(8,80) _= 12.87, ***p = 0.0005 **p < 0.0089 *p = 0.0143; when the *Ift52* siRNA group was compared with the control siRNA group; two-way repeated-measures ANOVA followed by Bonferroni’s multiple-comparisons test.

To independently validate the role of primary cilia in nociceptor function, we evaluated the effect of siRNA for a second intraflagellar protein, *Ift52*, in paclitaxel CIPN. Male rats were treated with siRNA targeting *Ift52* (10 μg, i.t., for 3 consecutive days) or negative control siRNA. Seventy-two hours after the last administration of *Ift52* siRNA, rats were killed and L4 and L5 DRGs harvested. Total RNA isolated from L4 and L5 DRGs was used to generate cDNA through reverse transcription. Quantitative RT-PCR performed upon the resultant cDNA revealed a significant decrease in *Ift52* expression in animals treated with siRNA targeting *Ift52* (Fig. 4*A*). We again employed paclitaxel CIPN as our neuropathic pain model, to evaluate the role of the nociceptor primary cilium in neuropathic pain. Male rats were treated with siRNA targeting *Ift52* in a dose of 10 μg/day (i.t.) for 3 consecutive days. Seventy-two hours after the last siRNA injection, paclitaxel (1 mg/kg, i.p.) was administered (Days 0, 2, 4, and 6). Mechanical nociceptive threshold was evaluated before siRNA treatment was started (baseline) and again on Days −3, −2, −1, 3, 5, 7, 14, 21, and 28 after the last dose of paclitaxel. Like the effect of *Ift88* siRNA, the siRNA targeting *Ift52* also reduced paclitaxel-induced hyperalgesia from Day 3 after the last administration of *Ift52* siRNA, as compared with rats receiving an intrathecal dose of a negative control siRNA (Fig. 4*B*,*C*). However, unlike Ift88 siRNA, Ift52 siRNA did not affect baseline mechanical nociceptive threshold (Fig. 4*B*).

### siRNA targeting *Ift88* attenuates PGE_2_-induced mechanical hyperalgesia

**Figure 5.**
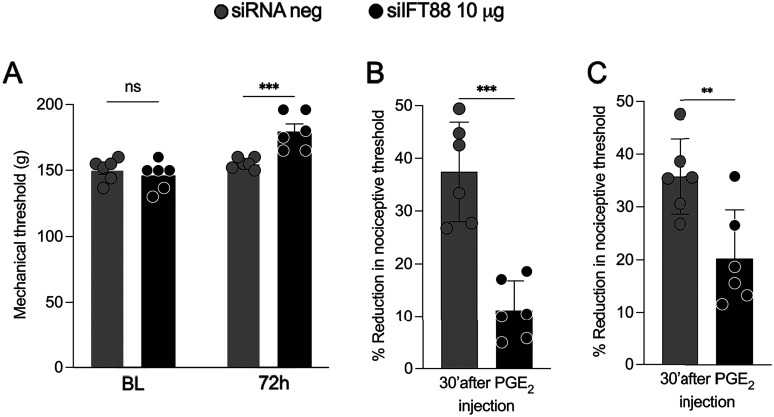
siRNA targeting *Ift88* attenuates PGE_2_-induced hyperalgesia. Male rats were treated with *Ift88* siRNA for 3 consecutive days, followed 72 and again 144 h later with PGE_2_ (100 ng/5 µl, i.d.), administered on the dorsal surface of one hindpaw. Mechanical nociceptive threshold was evaluated before treatment with siRNA (baseline), 72 h after the last siRNA treatment, and then 30 min after each of two sequential administrations of PGE_2_ separated by 72 h. ***A–C***, *Ift88* siRNA led to a significant increase in (***A***) baseline nociceptive threshold and (***B***, ***C***) reduction in PGE_2_ hyperalgesia, measured as % reduction from baseline mechanical nociceptive threshold (***B***) 72 h and then again (***C***) 144 h after the last treatment with siRNA.

We next examined the role of the primary cilium in a model of mechanical hyperalgesia induced by a clinically important inflammatory mediator, PGE_2_, a direct-acting pronociceptive inflammatory mediator (Pitchford and Levine, 1991; Gold et al., 1996; Hendrich et al., 2013) whose production is inhibited by the most important treatment for inflammatory pain, the nonsteroidal anti-inflammatory analgesics (NSAIDs). Rats were treated with siRNA targeting *Ift88* (10 μg/day, i.t.) or its negative control siRNA, for 3 consecutive days. Seventy-two hours after the final siRNA injection, PGE_2_ was administered on the dorsum of the hindpaw (100 ng/5 μl/paw, i.d.), at the site of nociceptive threshold testing. Nociceptive threshold was measured before siRNA treatment was started (baseline). Seventy-two hours after the last siRNA treatment, PGE_2_ was injected, and the magnitude of PGE_2_-induced hyperalgesia assessed 30 min later. Three days later PGE_2_ was again injected, and hyperalgesia assessed 30 post-PGE_2_ (Fig. 5). As was observed with baseline mechanical nociceptive threshold (Fig. 3*A*) in control rats, *Ift88* siRNA again led to an increase in the baseline nociceptive threshold (Fig. 5*A*). Treatment with *Ift88* siRNA also resulted in a marked reduction in PGE_2_-induced mechanical hyperalgesia, evaluated when PGE_2_ was injected 72 h after the last administration of *Ift88* siRNA and again 144 h later, to demonstrate persistence of the effect of *Ift88* siRNA (Fig. 5*B*,*C*).

### siRNA targeting *Ift88* attenuates PGE_2_-induced nociceptor sensitization, in vitro

**Figure 6.**
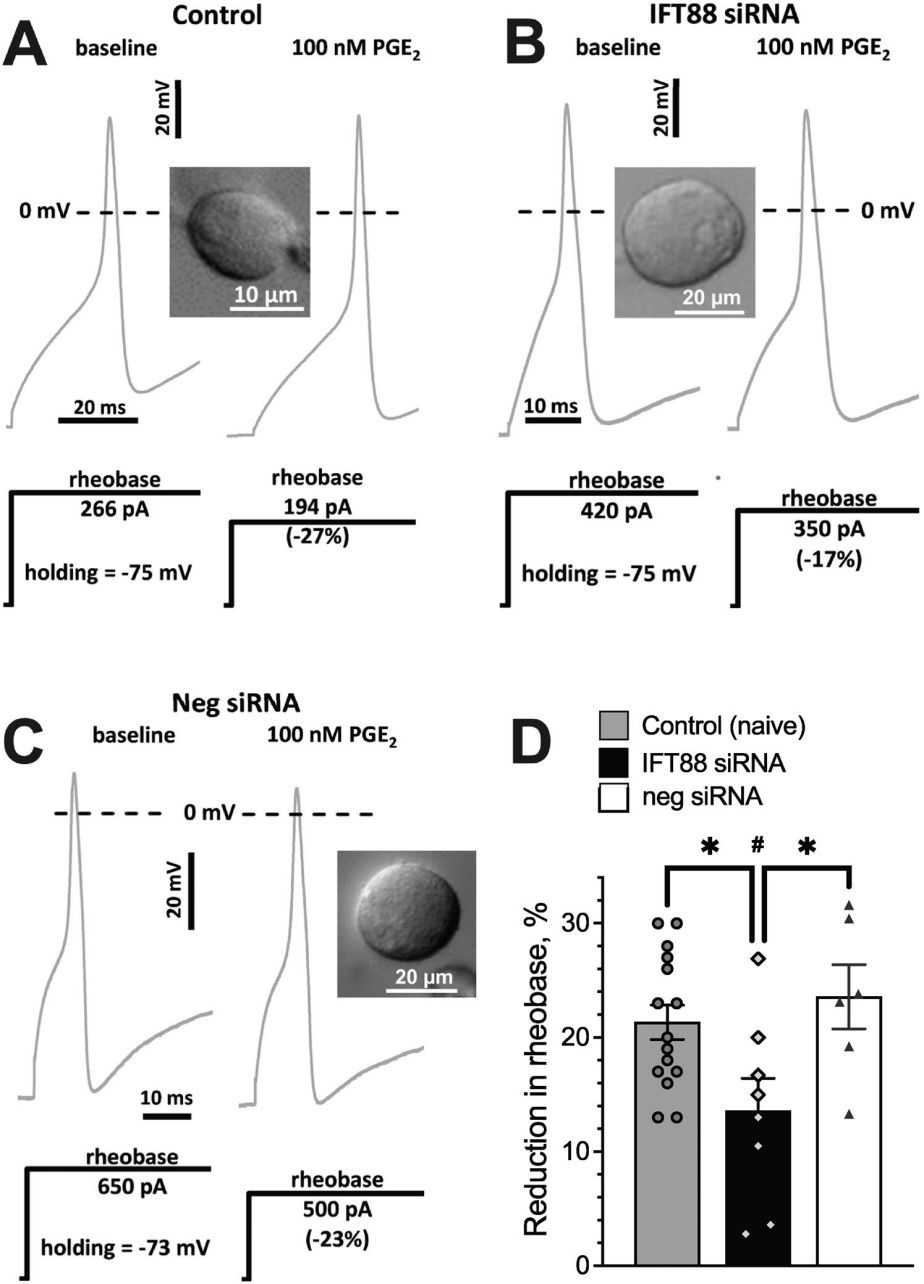
siRNA targeting *Ift88* increases action potential threshold, increasing peak outward currents, and attenuates PGE_2_-induced nociceptor sensitization. ***A–D***, PGE_2_-induced reduction of rheobase in putative C-type rat nociceptors cultured from control (***A***, naive), experimental (***B***, treated in vivo with siRNA targeting *Ift88*), and negative control (***C***, treated in vivo with negative siRNA) rats*.* Electrophysiological traces show APs generated in response to stimulation of small-diameter DRG neurons (inset) with a square wave current pulse (below AP recordings). The height of the pulse represents rheobase. Dotted line shows membrane potential of 0 mV. ***D***, Graphical representation of reduction in rheobase, comparing control rats to ones treated in vivo with *Ift88* siRNA and negative siRNA. Symbols show effect in individual neurons. *p = 0.02, *t*_(26) _= 2.6; ^#^*p *= 0.02, *t*_(26) _= 2.8, Šidák's multiple-comparisons test for ANOVA; effect of treatment is significant: *p *= 0.0017, *F*_(2,26) _= 4.8 (*n *= 15 control, 8 *Ift88* siRNA-treated and 6 negative siRNA-treated groups).

To test the hypothesis that the observed effect of *Ift88* siRNA on PGE_2_-induced hyperalgesia, in vivo, is nociceptor primary cilium dependent, we performed in vitro patch-clamp electrophysiology experiments, evaluating the effect of *Ift88* siRNA on PGE_2_*-*induced (100 nM) nociceptor sensitization, characterized by reduction in rheobase (Khomula et al., 2021) in small-diameter DRG neurons cultured from rats treated in vivo with *Ift88* siRNA and controls. Rheobase, the minimal sustained current required to generate an action potential (AP), a well-established electrophysiological property that reflects excitability in DRG neurons (Khomula et al., 2019, 2021; Vasylyev et al., 2023), was used to measure neuronal excitability. The percentage change in rheobase produced by PGE_2_ was evaluated in putative C-type nociceptive DRG neurons (soma diameter <35 µm; Harper and Lawson, 1985; Gold et al., 1996; Petruska et al., 2002; Woolf and Ma, 2007). PGE_2_ produced a reduction in rheobase in control nociceptors (Fig. 6*A*,*D*; 21.3 ± 1.5%; *n* = 15), and in neurons from rats treated with a negative control siRNA (Fig. 6*C*,*D*), while such a reduction in rheobase was significantly attenuated in *Ift88* siRNA-treated nociceptors (Fig. 6*B*,*D*; 13.6 ± 2.9%; *n* = 8; unpaired two-tailed Student's *t* test: *p = 0.015, *t*_(21) _= 2.66). These results support the suggestion that the sensitization of nociceptors by PGE_2_ is primary cilium dependent.

### Lack of effect of siRNA targeting *Ift88* on nociceptive threshold or PGE_2_ and CIPN hyperalgesia in female rats

**Figure 7.**
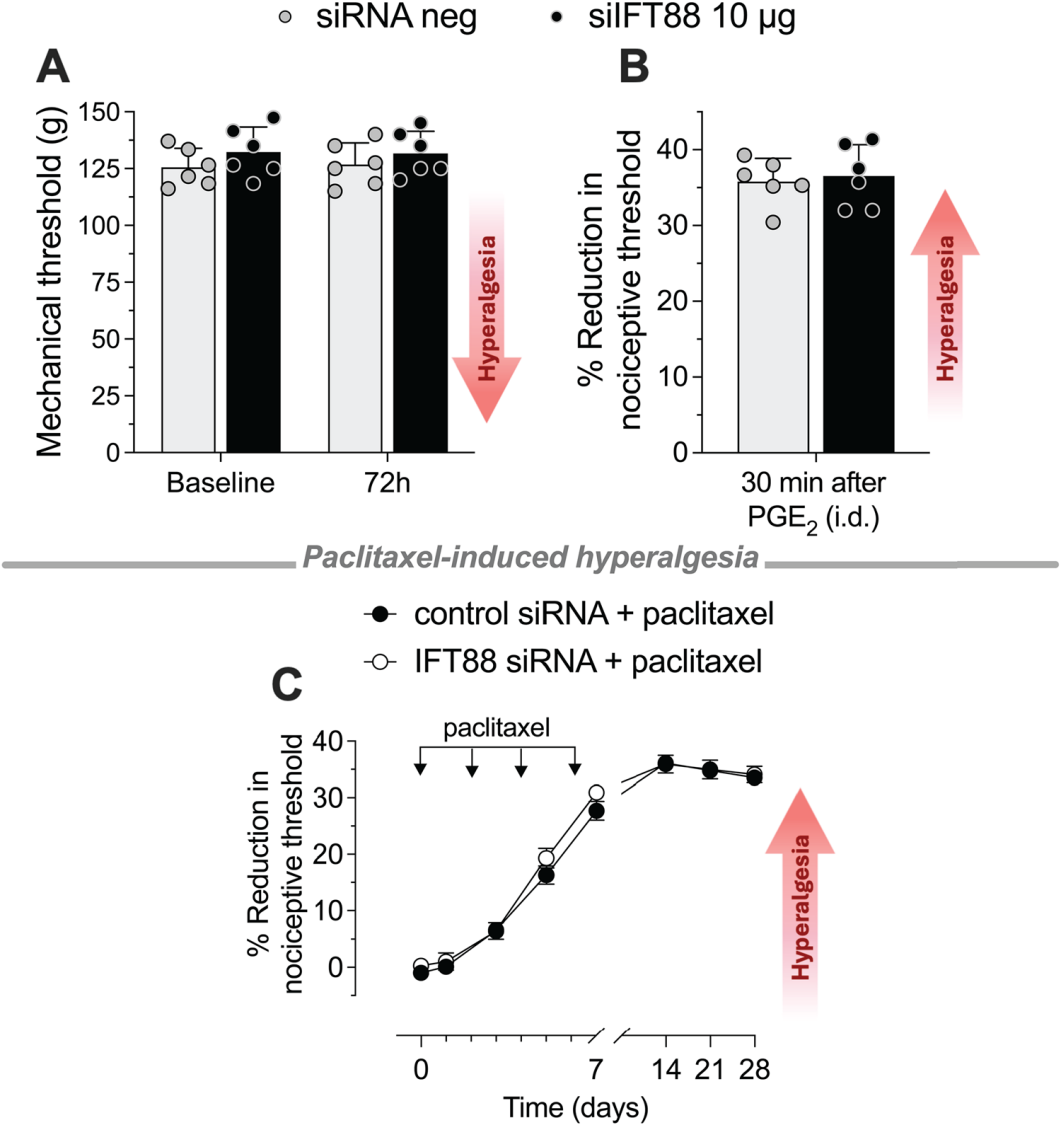
In female rats *Ift88* siRNA does not affect mechanical threshold and PGE_2_ or CIPN hyperalgesia. ***A***, Female rats were treated with siRNA for *Ift88* mRNA for 3 consecutive days in a dose of 10 µg/20 µl/day, intrathecally. Mechanical nociceptive threshold (in grams) was evaluated before siRNA treatment was started (baseline) and again 72 h after the last siRNA treatment. Measured 72 h after the last dose of siRNA targeting *Ift88*, there was no significant change in mechanical nociceptive threshold (data shown as means ± SEM, time *F*_(1,10) _= 0.1832, *p *= 0.6777: si*Ift88* vs siRNA neg control; two-way repeated-measures ANOVA followed by Bonferroni's multiple post hoc comparisons test, *n *= 6 paws of each group). ***B***, Seventy-two hours after the last siRNA injection, PGE_2_ was administered intradermally (100 ng/5 µl/paw, i.d.), and mechanical nociceptive threshold evaluated 30 min later; magnitude of hyperalgesia is expressed as percentage reduction from the baseline mechanical nociceptive threshold before siRNA treatment. In female rats, *Ift88* siRNA did not affect PGE_2_-induced hyperalgesia (data shown as mean ± SEM, unpaired Student's *t* test: *t *= 0.3576, df = 10; *p *= 0.7281: si*Ift88* vs siRNA neg control, *n* = 6 paws of each group). ***C***, Female rats were treated with siRNA targeting *Ift88* (10 µg/20 µl/day, i.t.) daily for 3 consecutive days or with its negative control siRNA (10 µg/20 µl/day). Seventy-two hours after the last injection of siRNA, paclitaxel was administered intraperitoneally (1 mg/kg, i.p.) on Days 0, 2, 4, and 6. Mechanical nociceptive threshold was evaluated before starting siRNA treatment (baseline), and again on Days 0 and 1, 3, 5, 7, 14, 21, and 28 after paclitaxel injection. Hyperalgesia induced by paclitaxel was unaffected in both the negative control and *Ift88* siRNA-treated groups (data are expressed as means ± SEM, *n *= 6 paws in each group; *F*_(1,10) _= 0.9413, *p *= 0.3548, when the *Ift88* siRNA group was compared with the negative control siRNA group; two-way repeated-measures ANOVA followed by Bonferroni's multiple post hoc comparisons test).

In non-neuronal cells, it is well established that the length of their primary cilium, which correlates with its function (Spasic and Jacobs, 2017; Macarelli et al., 2023), is estrogen dependent (Geoghegan et al., 2021; Hunter et al., 2024). Therefore, we determined if nociceptor primary cilia-dependent functions are sex dependent. To determine if the role of nociceptor primary cilia is sexually dimorphic, we investigated whether *Ift88* siRNA in female rats also increases nociceptive mechanical threshold and attenuates PGE_2_-induced hyperalgesia and paclitaxel-induced CIPN. In contrast to adult male rats, in adult females *Ift88* siRNA failed to increase nociceptive threshold (Fig. 7*A*) or attenuate either PGE_2_ hyperalgesia (Fig. 7*B*) or neuropathic CIPN hyperalgesia (Fig. 7*C*).

### Primary cilium dependence of the pronociceptive effect of Sonic hedgehog

**Figure 8.**
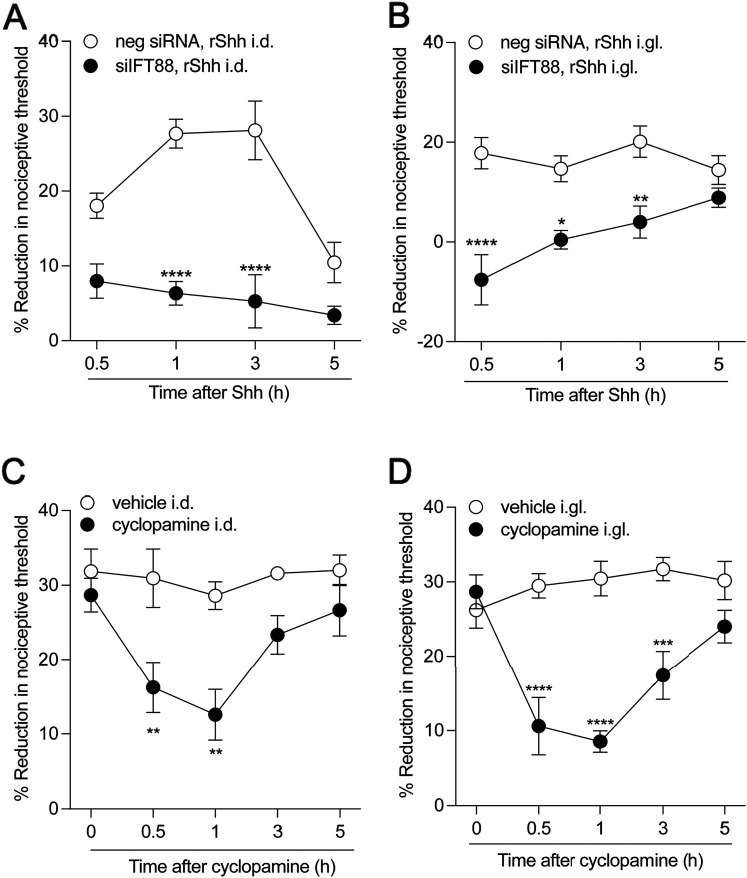
Pronociceptive effect of Shh is primary cilium dependent. ***A***, ***B***, Male rats were treated with siRNA targeting *Ift88* (10 µg/day, i.t.) for 3 consecutive days. Seventy-two hours after the last injection of siRNA, rats received intradermal or intraganglion injections of recombinant Shh (200 ng) and mechanical nociceptive threshold was assessed at 0.5, 1, 3, and 5 h after injection. ***A***, The hyperalgesic effect of Shh injected intradermally was significantly reduced by *Ift88* siRNA. Data shown as mean ± SEM, treatment *F*_(1,40)_ = 73.26, time *F*_(4,50) _= 6.861, ****p < 0.01: si*Ift88*, Shh intradermally versus siRNA neg, Shh intradermally. ***B***, The hyperalgesic effect of intraganglion Shh was also significantly reduced by *Ift88* siRNA. Data shown as mean ± SEM, treatment *F*_(1,40)_ = 48.13, time *F*_(3,40)_ = 2.286, ****p < 0.01: *Ift88* siRNA, Shh intraganglion versus siRNA neg, Shh intraganglion Two-way repeated-measures ANOVA followed by Bonferroni's test, *n* = 6 paws in each group. ***C***, ***D***, Male rats received paclitaxel every other day for a total of four injections, on Days 0, 2, 4, and 6 (1 mg/kg × 4, i.p.). Seven days after the first administration of paclitaxel, rats were treated with intradermal (10 µg/5 µl) or intraganglion (10 µg/5 µl) cyclopamine. As a control, the contralateral paw (i.d.) or contralateral lumbar DRG (i.gl.) received vehicle (saline plus DMSO 2%). Mechanical nociceptive threshold was evaluated before and 7 d after paclitaxel injection (before cyclopamine injection, 0 h), and again 0, 0.5, 1, 3, and 5 h after intradermal or intraganglion cyclopamine injection. ***C***, Paclitaxel-induced hyperalgesia was markedly attenuated in the male rats treated intradermally with cyclopamine. Data shown as mean ± SEM, treatment *F*_(1,50)_ = 28.28, time *F*_(4,50) _= 4.154, **p < 0.01: cyclopamine intradermal versus vehicle intradermal groups. ***D***, The magnitude of paclitaxel-induced hyperalgesia was markedly attenuated in male rats treated with intraganglion cyclopamine. Data shown as mean ± SEM, treatment *F*_(1,50)_ = 57.38, time *F*_(4,50) _= 4.748, **p < 0.01: cyclopamine intradermal versus vehicle intradermal.

While many effects of Hh are primary cilium dependent (Nozawa et al., 2013; Ho and Stearns, 2021), and Hh has been implicated in nociceptor sensitization (Babcock et al., 2011; Liu et al., 2018a, b; Han et al., 2020; Okuda et al., 2022; Zheng et al., 2023), the role of the primary cilium in the pronociceptive effects of Hh has not been explored. To test for dependence of pronociceptive effects of Shh on the primary cilium, male rats were treated with *Ift88* siRNA or its negative control, both in a dose of 10 μg/day, for 3 consecutive days. Seventy-two hours after administration of the last dose of *Ift88* siRNA, rats received an intradermal (Fig. 8*A*) or intraganglion (Fig. 8*B*) injection of recombinant Shh (200 ng), and nociceptive threshold was evaluated over time. Treatment of rats with *Ift88* siRNA reduced the hyperalgesia induced by both intradermal and intraganglion Shh (Fig. 8*A*,*B*). Of note, although nociceptive threshold was assessed by stimuli applied to the peripheral terminal of the nociceptor, the onset of hyperalgesia was more rapid when Shh was administered via the intraganglion route of administration.

To investigate the role of Hh signaling in nociceptor sensitization, in neuropathic pain, we again employed paclitaxel CIPN. Male rats were treated with paclitaxel (1 mg/kg × 4, i.p.) on Days 0, 2, 4, and 6. On Day 7, at which time primary cilium-dependent hyperalgesia (Figs. 3*E*,*F*, 4) was fully established, rats were treated with cyclopamine (10 μg) through intradermal (Fig. 8*C*) or intraganglion (Fig. 8*D*) injection, which inhibits Hh signaling by binding to the Smo coreceptor (J. K. Chen et al., 2002). Nociceptive threshold was evaluated 30 min and 1 and 3 h after cyclopamine treatment (*N* = 6). Administration of cyclopamine, by both routes of administration, attenuated paclitaxel-induced hyperalgesia (Fig. 8*C*,*D*).

### Selective siRNA-mediated knockdown of the *Drosophila Ift88* ortholog NompB specifically in nociceptors produces a hyposensitive mechanoceptive phenotype in *Drosophila* third instar larvae

**Figure 9.**
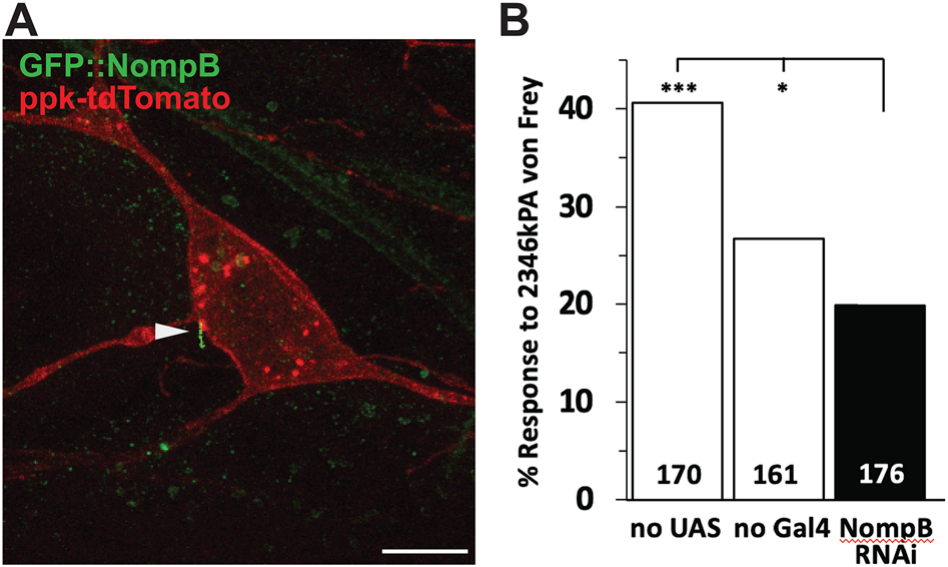
Nociceptor-targeted RNAi-mediated knockdown of the *Ift88* ortholog NompB in *Drosophila* larvae reduces sensitivity to noxious mechanical stimulation. ***A***, Immunohistofluorescence analysis of larval pickpocket-positive neurons in vivo. Fillet preparations of third instar ppk1.9-tdTomato larvae were labeled with antibodies recognizing a GFP::NompB fusion protein (GFP::NompB+; green). Pickpocket-expressing neurons (ppk/Td+) are indicated by Td-Tomato signal in red. White arrowhead indicates presumed neuronal primary cilium. Scale bar, 10 µm. ***B***, When third instar larvae of genotype ppk1.9-Gal4/UAS-NompB-RNAi were stimulated with a 2,346 kPa von Frey filament, the frequency of their escape response was significantly lower than that of normal controls “No UAS” (ppk1.9-Gal4/y^1^v^1^; *p* < 0.001) and “No Gal4” (w^1118^/UAS-NompB-RNAi; *p* < 0.05). *N* values (left to right) were 170, 161, and 176 animals. Response frequencies were compared by chi-square test.

NompB (Kernan et al., 1994), the *Drosophila* ortholog of *Ift88*, has been shown to be necessary for the growth of primary cilia in some sensory neurons (Type 1) of the fly (Y. G. Han et al., 2003), but its expression and function in nociceptors (Type 2, Class 4; Zhong et al., 2010) is not known. Genetically encoded fluorescent protein, immunohistofluorescence, and confocal microscopy techniques were employed to localize GFP-tagged NompB (Nagarkar-Jaiswal et al., 2015), revealing nociceptor-localized structures with dimensions and subcellular location consistent with those of primary cilia (Fig. 9*A*). We then tested the mechanical sensitivity of animals expressing a NompB RNAi element (Perkins et al., 2015) specifically in the nociceptor. Third instar *Drosophila* larvae bearing a NompB RNAi hairpin element under the control (Brand and Perrimon, 1993) of the nociceptor-specific *pickpocket* (Zhong et al., 2010) promoter were found to be significantly less sensitive to a midrange noxious mechanical stimulus (Lopez-Bellido et al., 2019) than normal controls (Fig. 9*B*). While over 40% of 170 “no UAS” and over 26% of 161 “no Gal4” normal control animals reacted with escape behavior in response to 2,346 kPA von Frey stimulation, <20% of 176 NompB RNAi animals reacted (*p* < 0.001 and *p* < 0.05, respectively; chi-square test; Fig. 9*B*).

## Discussion

Ciliation of DRG neurons has only recently been reported (Tan et al., 2007; Yusifov et al., 2021; W. W. Zhang et al., 2022). While two groups have reported that neurons from acutely dissociated DRG, prepared from adult mice, bear a primary cilium (Tan et al., 2007; W. W. Zhang et al., 2022), whether they are present in nociceptors had remained to be established. We have confirmed the presence of primary cilia on DRG neurons as well as shown, for the first time, based on cell body diameter (<35 μm) and the coexpression of characteristic proteins that ciliated DRG neurons include nociceptors, raising the possibility that primary cilia contribute to nociceptor function and may, therefore, play a role in baseline nociceptive threshold and inflammatory and neuropathic pain.

The primary cilium is often considered an antenna, acting as an integral part of its cell's sensory apparatus (Fitzsimons et al., 2022; DeMars et al., 2023; Mill et al., 2023). Therefore, after establishing that nociceptors express primary cilia, we tested the hypothesis that they are involved in the transduction of noxious stimuli and nociceptor sensitization as observed in inflammatory and neuropathic pain. We found that siRNA targeting *Ift88*, which is involved in the intraflagellar transport of proteins essential for ciliary structure and function (Pazour et al., 2000; Taulman et al., 2001); attenuates primary cilia in nociceptors; produces an elevation of mechanical nociceptive threshold and nociceptor rheobase, an in vitro measure of nociceptor threshold; and attenuates inflammatory and neuropathic pain, including PGE_2_-induced nociceptor sensitization, in vitro. However, the mechanism by which the primary cilium, located on the soma, contributes to the transduction of noxious mechanical stimuli and nociceptor sensitization, in the setting of inflammatory and neuropathic pain, at the nociceptor peripheral terminal, and whether the nociceptor primary cilium contributes to the detection of other noxious stimuli (e.g., noxious heat and cold, and pain produced by noxious chemicals such as capsaicin) remains unclear. As the central terminal of the nociceptor, located in the spinal dorsal horn, is involved in neurotransmission to second order neurons in pain circuitry, a possible role of primary cilia in pain neurotransmission, established for neurotransmission in the central nervous system (Sheu et al., 2022; Ott et al., 2023; Wang et al., 2024), should also be considered in future studies.

A precedent for primary cilium-dependent long-range control of biological events at remote terminal processes is afforded by studies examining embryonic axonal pathfinding. Axonal growth cones crossing the ventral midline of the spinal cord employ retrograde transport of midline-derived Shh for activation of Hh signaling cascades at the primary cilium on the cell body of the same neuron, hundreds of cell diameters away (Dumoulin et al., 2024). Similarly, retinal ganglion cells have been reported to use anterograde transport of Shh to the optic chiasm, where secretion could direct axonal outgrowth (Peng et al., 2018). However, the timeframe of nociceptive modulation seen following peripheral application of Shh and cyclopamine in the present experiments would seemingly preclude the involvement of such retrograde signaling. Alternatively Shh enhances calcium oscillations in embryonic neurons (Belgacem and Borodinsky, 2011). Calcium signaling can propagate rapidly along axons (Cho et al., 2013; Saito and Cavalli, 2016), and primary cilia are specialized calcium signaling cellular organelles (Kleene, 2022; Sanchez et al., 2023; Vien et al., 2023). Such dependence of the function of primary cilia in nociceptor function on short- and long-range calcium signaling remains to be established.

Nociceptors are sensitized by diverse inflammatory mediators to produce mechanical hyperalgesia. Among these are prostaglandins, whose synthesis is inhibited by NSAIDs, the most commonly used analgesics for the treatment of inflammatory pain. We determined if primary cilia contribute to PGE_2_-induced nociceptor sensitization. Treatment of rats with *Ift88* siRNA markedly attenuated the mechanical hyperalgesia induced by PGE_2_. To confirm that this effect of *Ift88* siRNA was mediated by its effect on nociceptors, we performed patch-clamp electrophysiology on DRG neurons cultured from rats treated with *Ift88* siRNA. In these nociceptors, rheobase was elevated and the PGE_2_-induced decrease in rheobase attenuated, providing direct support that the elevation of nociceptive threshold and attenuation of PGE_2_-induced hyperalgesia observed in vivo is dependent on the effect of *Ift88* siRNA on nociceptors. However, how primary cilia, which are located at the nociceptor cell body and contain elements of signaling pathways implicated in nociceptor sensitization (e.g., adenylyl cyclase; Qiu et al., 2016; Tschaikner et al., 2020; Paolocci and Zaccolo, 2023), contribute to nociceptor sensitization at their peripheral terminals remains unclear.

Since sensitized nociceptors play an important role in humans (Binder et al., 2007; Serra et al., 2012; Uceyler, 2016; Landmann et al., 2022) and animals (Tanner et al., 1998; Dina et al., 2001; Tanner et al., 2003; X. Chen et al., 2011; Villalba-Riquelme et al., 2022) with diverse forms of neuropathic pain, we also evaluated whether CIPN, induced by a chemotherapy drug, paclitaxel, is primary cilium dependent. In support of this hypothesis, we found that siRNA targeting *Ift88* markedly attenuates CIPN.

To provide independent confirmation of the role of the primary cilium in nociceptor function, we evaluated its dependence on the contribution of a second intraflagellar protein, IFT52. As observed following treatment with *Ift88* siRNA, *Ift52* siRNA also markedly attenuated paclitaxel CIPN. However, unlike *Ift88* siRNA, which produced an elevation of nociceptive threshold as well as attenuation of CIPN, *Ift52* siRNA did not affect baseline nociceptive threshold. While details of the underlying mechanism by which primary cilium signaling communicates with terminals of nociceptive sensory neurons remains to be elucidated, our results support a role of the primary cilium both in setting baseline mechanical nociceptive (by IFT88) threshold and nociceptor sensitization (by IFT88 and IFT52).

The primary cilium contains many receptors, second messengers and ion channels (Kobayashi et al., 2020; Wachten and Mick, 2021; Paolocci and Zaccolo, 2023), some of which have been implicated in nociceptor function (Duffy et al., 2021; Arena and Hofer, 2022; Fagan et al., 2024). One such primary cilium-dependent signaling pathway is Hh. Thus, it has been shown that selective inhibitors of Hh signaling (e.g., vismodegib and cyclopamine) attenuate mechanical hyperalgesia associated with inflammation (Babcock et al., 2011; Yang et al., 2020) and peripheral neuropathy (Okuda et al., 2022), whereas activation of Hh signaling (e.g., with SAG, a smoothened agonist) produces hyperalgesia (Yang et al., 2020; Okuda et al., 2022). We demonstrate that the hyperalgesia induced by recombinant Shh is markedly attenuated in rats treated with *Ift88* siRNA. Of note, however, in contrast to the effect of *Ift88* siRNA, which increased baseline mechanical nociceptive threshold, inhibitors of Hh signaling, in the absence of inflammation or peripheral neuropathy, did not affect nociceptive threshold, supporting the presence of an Hh-independent primary cilium function capable of regulating baseline mechanical nociceptive threshold.

Another primary cilium-dependent cellular signaling pathway that has been found to contribute to nociception is the wingless-related integration site (Wnt)/β-catenin signaling pathway (Kim et al., 2021; Damo and Simonetti, 2022; Hale et al., 2022; Lu et al., 2022; Tang et al., 2022; Zhou et al., 2022; Bai et al., 2023). Ciliary proteins are not only necessary for certain types of Wnt signaling but also include effectors downstream of Wnt (Lee, 2020; Karalis et al., 2022; Yin et al., 2022; K. Zhang et al., 2023). Of note, inhibitors of Wnt/β-catenin signaling have been reported to attenuate CIPN (Resham and Sharma, 2019; Diamond et al., 2020; Hu et al., 2020), and Wnt has been shown to enhance neuronal excitability (Simonetti et al., 2014; Simonetti, 2015). WNT agonists directly generate hypernociception (Yuan et al., 2015) and Wnt3a recruits Wnt-calcium signaling in sensory neurons to enhance pain sensitivity (Simonetti et al., 2014; Liu et al., 2015). In contrast to Shh signaling, the fly homolog of β-catenin, Armadillo, does regulate baseline nociceptor sensitivity (Hale et al., 2022).

In summary, we established that primary cilia are present on adult nociceptors and that siRNA coding for *Ift88*, an intraflagellar protein essential for the integrity of the primary cilium, attenuates primary cilia in nociceptors. *Ift88* siRNA produced a phenotype that includes increased mechanical nociceptive threshold and attenuation of inflammatory and neuropathic pain. Using electrophysiology on nociceptors cultured from rat DRG, we confirmed that *Ift88* siRNA increases rheobase, by selectively increasing peak outward current, and attenuates PGE_2_-induced nociceptor sensitization, an in vitro measure of hyperalgesia. To confirm the contribution of the primary cilium to the pain phenotype produced by siRNA targeting *Ift88*, we established a pain phenotype with siRNA targeting *Ift52*. Unexpectedly, *Ift88* siRNA did not affect baseline nociceptive threshold and inflammatory and neuropathic pain in female rats, a finding requiring extensive additional studies to determine the length and function of the neuronal primary cilium in the female nociceptor, and whether differences in the role of the primary cilium in male and female nociceptors is female sex hormone dependent. Finally, our observation that nociceptor-specific RNA interference targeting the *Ift88* ortholog NompB reduces mechanical sensitivity in *Drosophila* supports the notion that the control of pain sensitivity by primary cilia may be a mechanism that is conserved across Metazoa and perhaps as ancient as the nervous system itself. A major unresolved issue is how the primary cilium which is localized to the neuronal soma regulates the function of its remotely located terminals.
