# Supplementary figures and images for: The Nociceptor Primary Cilium Contributes to Mechanical Nociceptive Threshold and Inflammatory and Neuropathic Pain
Source: J Neurosci. 2024 Sep 30;44(47):e1265242024. doi: 10.1523/JNEUROSCI.1265-24.2024 (PMC11580782; doi:10.1523/JNEUROSCI.1265-24.2024)

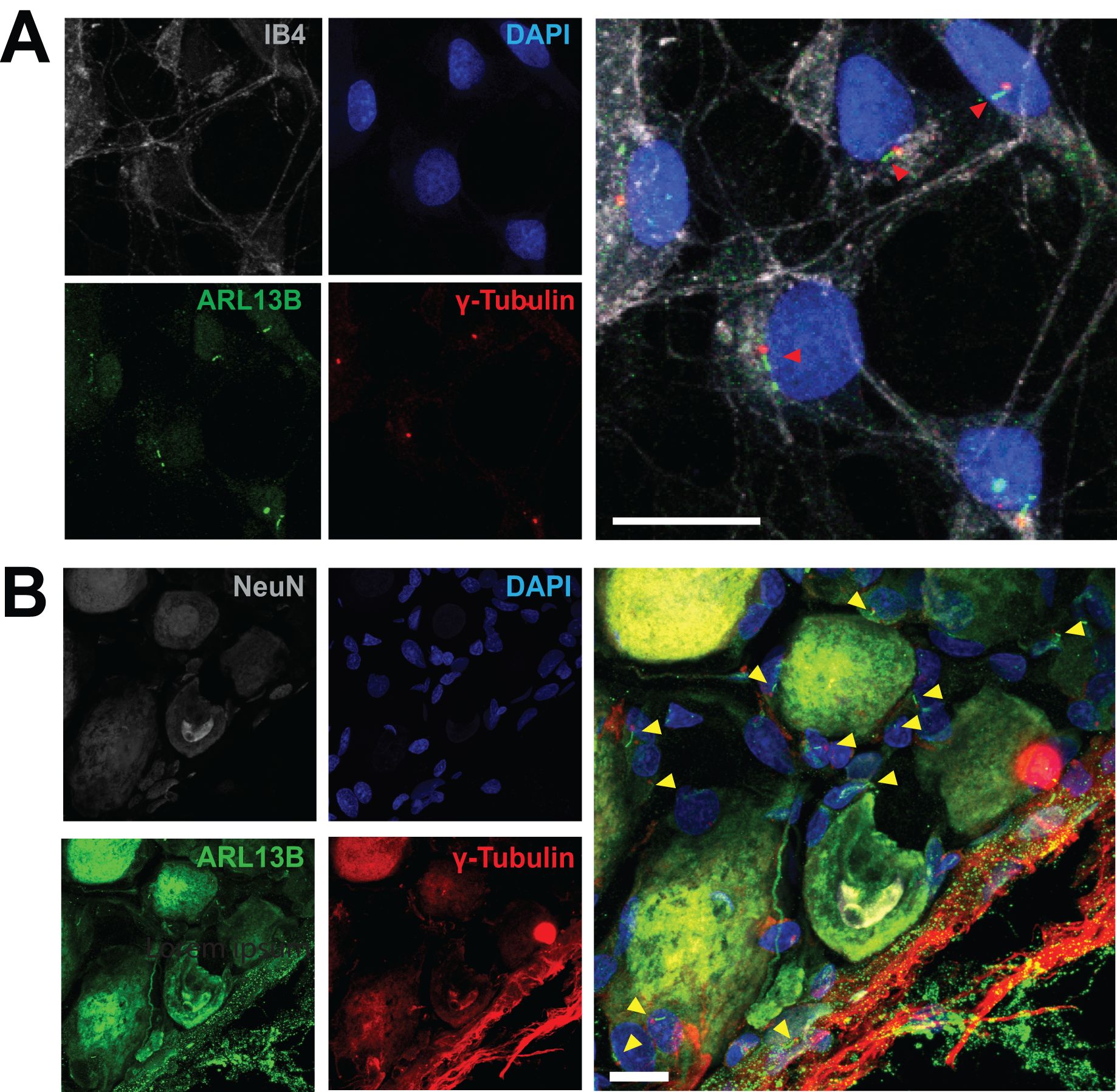

Supplement: Figure 1-1 — Rat DRG neurons elaborate primary cilia in vitro and in vivo. (A, B) Immunohistofluorescence analysis of acutely dissociated cultured adult rat DRG (A) and of adult rat DRGs in vivo (B). Coverslips (A) and histological sections (B) were labeled with antibodies recognizing ARL13B (green), ɣ-tubulin (red), and Fox-3 (B, NeuN, greyscale). (A) IB4 staining is indicated in greyscale. (A, B) Cell nuclei marked by DAPI (blue). Red and yellow arrowheads indicate neuronal and non-neuronal primary cilia, respectively. Scale bar: 10 µm. Download Figure 1-1, TIF file. [file jneuro-44-e1265242024-s001.tif]
